# Identification of two novel ferroptosis-associated targets in sepsis-induced cardiac injury: Hmox1 and Slc7a11

**DOI:** 10.3389/fcvm.2023.1185924

**Published:** 2023-06-23

**Authors:** Yushun Xu, Gang Bu

**Affiliations:** ^1^ Department of Cardiology, Taizhou Central Hospital (Taizhou University Hospital), Taizhou, China; ^2^ Department of Cardiology, The Second Affiliated Hospital of Zhejiang Chinese Medical University, Hangzhou, China

**Keywords:** ferroptosis, targets, sepsis, cardiac injury, bioinformatics

## Abstract

**Purpose:**

Sepsis-induced cardiac injury is a severe complication of sepsis and has a high mortality. Recent research has implicated ferroptosis as a contributing factor to myocardial cell death. This study is aimed at finding novel ferroptosis-associated targets in sepsis-induced cardiac injury.

**Methods and results:**

In our study, a total of two Gene expression omnibus datasets (GSE185754 and GSE171546) were obtained for bioinformatics analysis. GSEA enrichment analysis demonstrated that ferroptosis pathway Z-score rapidly increased in the first 24 h and decreased gradually in the following 24–72 h. Fuzzy analysis was then used to obtain distinct clusters of temporal patterns and find genes in cluster 4 that exhibited the same trend with ferroptosis progression during the time points. After intersecting the differentially expressed genes, genes in cluster 4, and ferroptosis-related genes, three ferroptosis-associated targets were finally selected: Ptgs2, Hmox1, and Slc7a11. While Ptgs2 has been previously reported to be involved in the regulation of septic cardiomyopathy, this study is the first to demonstrate that downregulation of Hmox1 and Slc7a11 can alleviate ferroptosis in sepsis-induced cardiac injury.

**Conclusion:**

This study reports Hmox1 and Slc7a11 as ferroptosis-associated targets in sepsis-induced cardiac injury, and both of them may become key therapeutic and diagnostic targets for this complication in the future.

## 1. Introduction

Sepsis is a kind of systemic inflammatory response that triggers excessive production of reactive oxygen species (ROS), elevated levels of proinflammatory cytokines, and extensive oxidative stress, and finally results in multiple organ dysfunction (1). Generally, sepsis-induced myocardial injury is a pervasive complication observed in patients with sepsis. The incidence rate of cardiac dysfunction within 3 days after the onset of sepsis is nearly 60% and the mortality rate has been reported to be as high as nearly 80% (2–4). Several mechanisms have already been suggested to elucidate the complicated pathophysiological progress of sepsis-induced cardiac injury, including the alteration of calcium ion homeostasis, depletion of energy, and change of oxidative stress, which all terminally lead to the death of differentiated cardiomyocytes (5, 6). However, the current treatments for sepsis-induced cardiac injury continue to be unsatisfactory, indicating that there is still a lack of definite molecular mechanisms and specific targets for sepsis-induced cardiac injury.

Myocardial cell death has been recognized as a crucial pathogenic characteristic during the progression of cardiac injury. In recent years, an increasing number of reports have highlighted the involvement of ferroptosis, a novel form of regulated cell death, in sepsis-induced cardiac injury (7). In ferroptosis, a redox imbalance has been created on the basis of the accumulation of lipid oxidation and free radicals produced by abnormal enzymes (8). Excessive ROS is generated by intracellular iron through Fenton reaction, which occurs on lipid membranes and leads to ferroptosis (9, 10). Previous studies revealed that lipopolysaccharide (LPS)-treated models with cardiac injury elevated the expression of prostaglandin endoperoxide synthase 2 (Ptgs2), a recognized marker of ferroptosis (7, 11). Meanwhile, Ferrostatin-1 (Fer-1), an acknowledged ferroptosis inhibitor, was also found to improve sepsis-induced cardiac injury significantly via the TLR4/NF-*κ*B signaling pathway (11). Existing evidence has indicated the vital position of ferroptosis in regulating sepsis-induced cardiac dysfunction. This study is aimed at investigating novel ferroptosis-associated targets in sepsis-induced cardiac injury in order to provide inspiring targets in clinical use for the treatment and diagnosis of sepsis. Bioinformatics analysis was conducted for identifying the differentially expressed genes (DEGs) of sepsis based on GSE185754 and GSE171546 datasets. After intersecting with the ferroptosis-related genes (FRGs), a total of three ferroptosis-associated targets, Ptgs2, solute carrier family 7 member 11 (Slc7a11), and heme oxygenase 1 (Hmox1), were obtained. Ptgs2 has been shown to be involved in the regulation of septic cardiomyopathy, and SLC7A11 expression has been shown to be significantly elevated in LPS-treated BEAS-2B (human bronchial epithelial cells) and lung tissues, but restored through Fer-1 (10–12). In addition, there have been reports indicating the involvement of Hmox1 in the regulation of iron-mediated cell death in sepsis (13).Therefore, LPS-treated mice and primary rat cardiomyocytes were used to confirm the expression and function of targets related to iron poisoning.

## 2. Materials and methods

### 2.1. Data selection

The RNA expression data of sepsis-induced cardiac injury were obtained by retrieval throughout the Gene expression omnibus (GEO) database (http://www.ncbi.nlm.nih.gov/geo/). A total of two datasets, GSE185754 and GSE171546, were obtained for analysis by using “sepsis” and “cardiac injury” as key words. In GSE185754 (GPL24247, Illumina NovaSeq 6,000), cardiac mRNA profiles of five saline-injected control C57BL/6J mice, and five LPS-stressed 24 h mice were acquired. GSE171546 (GPL24247, Illumina NovaSeq 6,000) contained cardiac mRNA profiles of C57BL/6J mice after cecal ligation and puncture (CLP) for 24, 48, and 72 h. A total of two datasets, GSE215955 and GSE53007, were further used to validate the expression of ferroptosis-associated targets. In GSE215955 (GPL21273, HiSeq X Ten), RNA sequencing was performed in the heart of C57BL/6 mice from the control and LPS-induced heart injury group (*n* = 4 for each group). In GSE53007 (GPL6885, Illumina MouseRef-8 v2.0 expression beadchip), RNA was extracted from the murine heart muscle tissues (*n* = 4 for sepsis, *n* = 4 for control) and subsequently microarray analysis was performed to compare the transcriptomic responses.

### 2.2. Differential expression analysis

The DESeq2 package was used to identify DEGs in cardiac mRNA profiles between sepsis and control groups. The primary method for normalization in DESeq2 involved the regularized-logarithm transformation based on reads per million mapped reads (RPM), which facilitated the calculation of a scaling factor for each sample. RPM = Number of reads mapped to a gene × 10^6^/Total number of mapped reads from the given library. The distribution of read counts in GSE185754 and GSE171546 can be seen in Supplementary Figure S1. The DEGs were determined as |log2-fold change (FC)| > 1 and *P* < 0.05. “clusterProfiler” and “enrichplot” were packages used for Gene Ontology (GO) enrichment analysis and Kyoto Encyclopedia of Genes and Genomes (KEGG) pathway enrichment analysis, respectively.

### 2.3. GSEA enrichment analysis

To investigate ferroptosis in the progress of sepsis-induced cardiac dysfunction, all ferroptosis pathway genes from KEGG (mmu04216) were downloaded from the KEGG database. Enrichment analysis was performed on GSE185754 and GSE171546 using GSEA 4.1.0. The Ferroptosis pathway *Z*-score of each sample in GSE185754 and GSE171546 was obtained by using the GSVA ssGSEA method, and statistical differences between the groups were confirmed.

### 2.4. Fuzzy C-means clustering

As a clustering method for processing gene profiling data, Mfuzz package was used to analyze GSE171546 for grouping DEGs into different clusters (14). The core algorithm of “Mfuzz” based on Fuzzy C-Means Clustering (FCM) was specifically developed for analyzing the time trend of gene expression and cluster genes with similar expression patterns to help understand the dynamic patterns of these biological molecules and their connection with functions.

### 2.5. Animals and study design

All procedures in our study on animals were approved by the Institutional Animal Care and Use Committee, Zhejiang Center of Laboratory Animals (IACUC, ZJCLA) (Approval No. ZJCLA-IACUC-20050030). All newborn Sprague Dawley (SD) rats (5–6 g) and C57BL/6 mice (30–40 g) were purchased from the Hangzhou Medical College Laboratory Animal Center (Hangzhou, China) and conditioned with sterile water and sufficient feed following standard laboratory conditions for 1 week before modeling. The sepsis-induced cardiac injury model was created by the injection of LPS dissolved in clear saline (10 mg/kg) for 1 week (15). 24 mice were randomly divided into control group (*n* = 6), LPS group (*n* = 6), LPS + Fer-1 group (*n* = 6) and RSL group (*n* = 6) by simple random sampling. The mice were injected with LPS, Fer-1 (Sigma, USA), and RSL (MCE, USA) and intraperitoneally injected with Fer-1 at a dose of 1 mg/kg for 1 week (7) and RSL at a dose of 100 mg/kg twice a week for 20 days (7). The mice were fed with normal diet for 1 week and then euthanatized with CO_2_ on day 7 (*n* = 6 in each group) for the collection of heart tissues. In order to further analyze the time-course expression of genes, a total of 24 mice were treated intraperitoneally with LPS (10 mg/kg) for 1 week to create the model of sepsis-induced cardiac injury. After successful modeling, every 6 mice were euthanatized every other day (0-hour group: euthanasia on D7, 24 h group: euthanasia on D8, 48 h group: euthanasia on D9, 72 h group: euthanasia on D10). We also purchased five newborn SD rats (1–3 days) and fed them for 2 days. Then, the rats were euthanized by intraperitoneal injection with 10% chloral hydrate and their heart tissues were collected for further collection of myocardial cells. The complete ARRIVE Guidelines checklist can be found in the Supplemental material.

### 2.6. Confirmation of animal models

Echocardiography was performed by utilizing a linear array ultrasound transducer (10-MHz, Biosound Esaote, Italy). Following the administration of 1.5% isoflurane for anesthesia, the averaged parameters such as heart rate (bpm), ejection fraction%, and fraction shortening% were recorded from three to five cardiac cycles for each group. Ejection fraction% is a measurement of the percentage of blood leaving heart each time it contracts. Fractional shortening% quantifies the decrease in the length of the end-diastolic diameter that occurs by the end of systole.

### 2.7. Cardiac tissue staining

Cardiac tissue sections collected from the mice were deparaffinized and hydrated in distilled water. Lillie's ferric iron stain kit (Solarbio, China) was applied for Prussian blue staining to detect the presence of ferric iron in the tissues. Working iron staining was created on the basis of the mixture of hydrochloric acid solution and potassium ferrocyanide solution. All the slides were incubated in a working iron stain solution and a nuclear fast red solution successively according to relevant instructions. Hematoxylin and eosin (H&E) staining was conducted as per the protocol of the H&E Staining Kit (Solarbio, China). All the slides were observed by using a light microscope (Olympus, Japan). DCF-cellular ROS assay (Abcam, United Kingdom) was also used to assess the ROS level in the cardiac tissue samples quantitatively.

### 2.8. Cell culture and treatment

Rat myocardial cells were isolated from newborn SD rats (1–3 days) according to previous reports (16, 17). The isolated rat myocardial primary cells were seeded in six-well plates and cultured in Dulbecco's modified Eagle's medium (DMEM) containing 10% fetal bovine serum (FBS) and 1% penicillin/streptomycin. Immunofluorescent staining was also carried out for the evaluation of cardiomyocytes. To validate the functions of ferroptosis-associated targets, the primary myocardial cells were transfected with Ptgs2 siRNA, Hmox1 siRNA, and Slc7a11 siRNA using Lipofectamine 3000 (Invitrogen, China) according to the manufacturer's protocol for 24 h until the cell confluence reached approximately 70%. The morphology of the cells was examined under a light microscope after 48 h of treatment and images of the cell morphology were taken.

### 2.9. Cell viability

To determine the viability of cells, we placed 6 × 10^3^ cells per well of a 96-well plate. The CCK-8 kit (Dojindo, Japan) was used to monitor cell viability following the suggestions of the producers. The viability of cells was calculated according to the protocol.

### 2.10. RNA extraction and qRT-PCR

The TRIzol reagent (Invitrogen, United States) was used for extraction of RNA from the collected cardiac tissues. The reverse transcription of RNA into cDNA was performed using the Reverse Transcription Kit (Takara, China). The SYBR Green kit (Takara, China) was applied for carrying out real-time PCR analysis as per the instructions. The primers of Ptgs2, Hmox1, and Slc7a11 used in qRT-PCR are listed in Table 1.

**Table 1:** The primers of Ptgs2, Hmox1, and Slc7a11.

Name	Forward	Reverse
Ptgs2	ATTCCAAACCAGCAGACTCATA	CTTGAGTTTGAAGTGGTAACCG
Hmox1	AGGTACACATCCAAGCCGAGA	CATCACCAGCTTAAAGCCTTCT
Slc7a11	CTATTTTACCACCATCAGTGCG	AATCGGGACTGCTAATGAGAATT
Gapdh	GGCAAATTCAACGGCACAGTCAAG	TCGCTCCTGGAAGATGGTGATGG

Ptgs2, prostaglandin endoperoxide synthase 2; Slc7a11, solute carrier family 7 member 11; Hmox1, heme oxygenase 1.

### 2.11. Statistical analysis

The entire statistical analyses were conducted using the R software (version 3.6.5). Gene-enriched signaling and process was determined by using Fisher's exact test and DAVID functional annotation. The experiments were repeated a minimum of three times. To evaluate the cardiac functional parameters among the three groups, a one-way analysis of variance (ANOVA) was initially conducted. Subsequently, if necessary, *post hoc* analysis with Bonferroni correction was performed for multiple comparisons. To confirm the statistical significance of differences in different groups, Student's *t*-test and the one-way ANOVA was also performed, and *P* < 0.05 was considered statistically significant.

## 3. Results

### 3.1. Identification of DEGs in sepsis-induced cardiac injury

**Figure 1:**
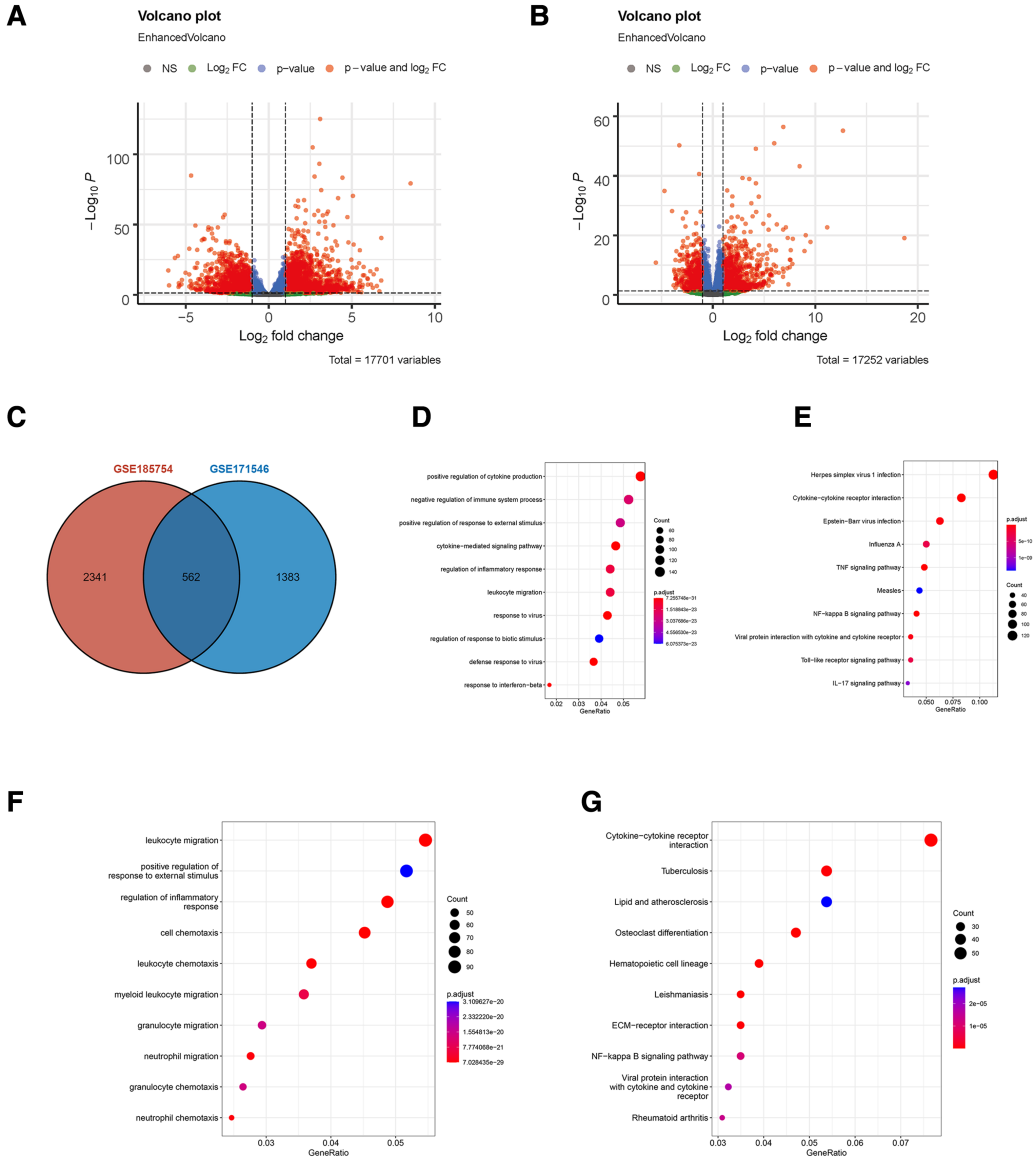
Identification of DEGs in sepsis-induced cardiac injury. (**A**) Volcano plot of DEGs in GSE185754. (**B**) Volcano plot of DEGs in GSE171546. The red plots represent DEGs with *P* < 0.05 and |log2-fold change (FC)| > 1 and *P* < 0.05. (**C**) The intersection of DEGs from both GSE185754 and GSE171546. (**D**) Functional enrichment analysis of DEGs from GSE185754. Top 10 terms in GO analysis with *P* < 0.05. (**E**) Top 10 terms in the KEGG pathway analysis of DEGs from GSE185754 with *P* < 0.05. (**F**) Functional enrichment analysis of DEGs from GSE171546. Top 10 terms in GO analysis with *P* < 0.05. (**G**) Top 10 terms in the KEGG pathway analysis of DEGs from GSE171546 with *P* < 0.05. DEGs, differentially expressed genes; GO, Gene Ontology; KEGG, Kyoto Encyclopedia of Genes and Genomes.

A total of two RNA profiling expressions, GSE185754 and GSE171546, were downloaded for analysis. A series of strict filters were carried out to provide different expression levels of genes from the sepsis-induced cardiac injury group and the normal group. A volcano analysis showed 2,903 DEGs obtained from GSE185754, including 1,574 upregulated DEGs and 1,329 downregulated DEGs (Figure 1A), and 1,945 DEGs obtained from GSE171546, including 1,125 upregulated DEGs and 820 downregulated DEGs (Figure 1B). A total of 562 DEGs were observed in both GSE185754 and GSE171546 (Figure 1C). The enrichment analysis showed that DEGs from GSE185754 were enriched in GO terms containing a positive regulation of cytokine production, a cytokine-mediated signaling pathway, and a regulation of inflammatory responses (Figure 1D). KEGG enrichment analysis showed that all DEGs from GSE185754 were enriched in the TNF signaling pathway, cytokine–cytokine receptor interaction, and herpes simplex virus 1 infection (Figure 1E). GO and KEGG enrichment analysis of the DEGs from GSE171546 also revealed significant enrichment in leukocyte migration, a positive regulation of response to external stimulus, and cytokine–cytokine receptor interaction (Figures 1F,G).

### 3.2. Ferroptosis pathway in enrichment analysis

**Figure 2:**
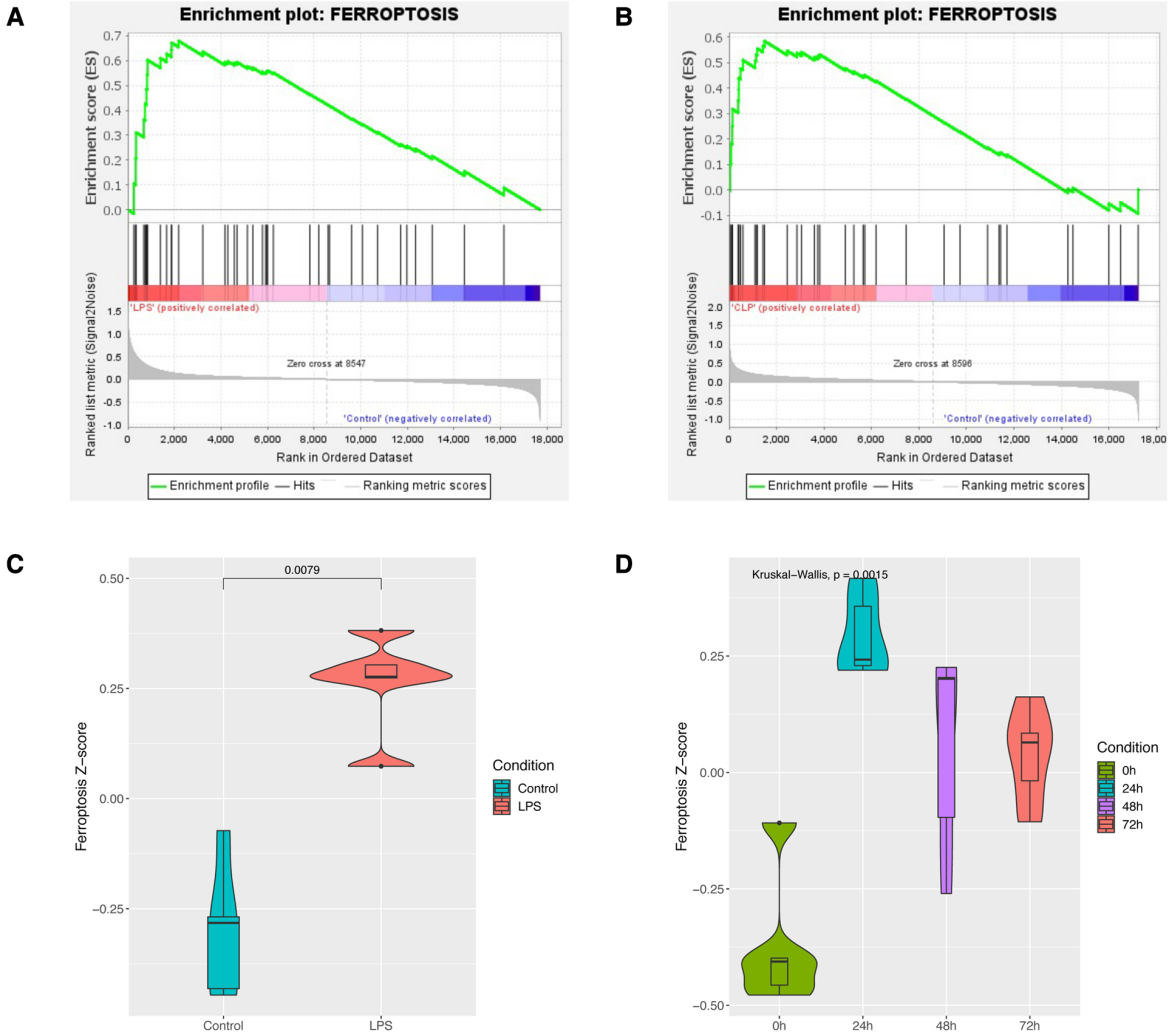
Enrichment analysis of the ferroptosis pathway in sepsis-induced cardiac injury. (**A**) GSEA enrichment analysis of the ferroptosis pathway in GSE185754. (**B**) GSEA enrichment analysis of the ferroptosis pathway in GSE171546. (**C**) The ferroptosis pathway Z-score of GSE185754. (**D**) The ferroptosis pathway Z-score of GSE171546.

GSEA enrichment analysis showed that the sepsis-induced cardiac injury group was significantly enriched in the ferroptosis pathway in both GSE185754 and GSE171546 (Figures 2A,B). ssGSEA analysis also showed that the ferroptosis pathway *Z*-score of the GSE185754 LPS group was significantly higher than that of the control group (Figure 2C), and the ferroptosis pathway *Z*-score of the CLP group in GSE171546 was significantly higher than that of the control group. Interestingly, the ferroptosis pathway *Z*-score increased rapidly in the first 24 h after CLP and reached a peak in 24 h, and then gradually decreased from 24 to 72 h (Figure 2D).

### 3.3. Fuzzy analysis of GSE171546

**Figure 3:**
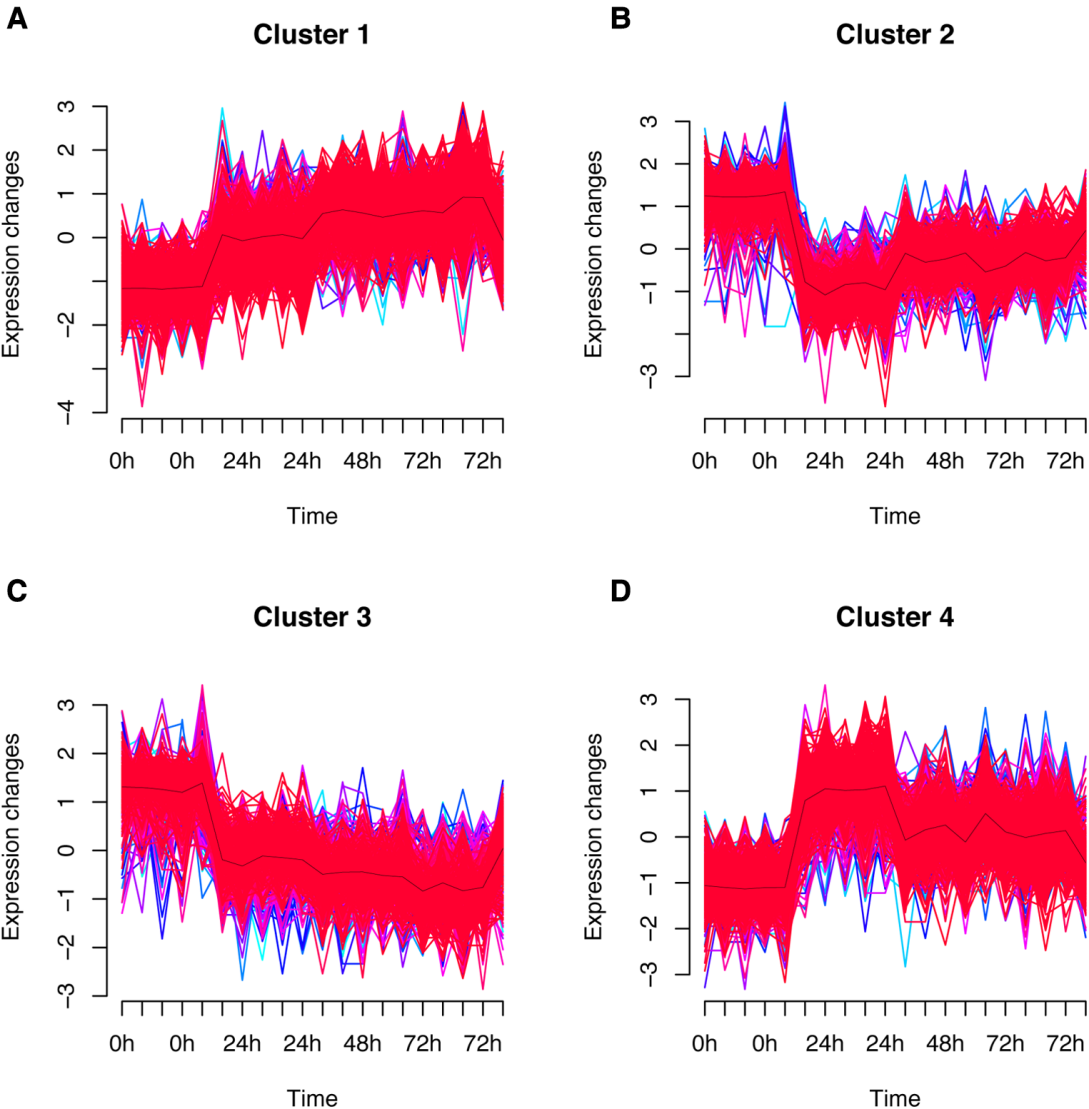
Fuzzy analysis of GSE171546. The fuzzy c-means algorithm was applied to cluster gene expression profiles in cluster1 (**A**), cluster2 (**B**), cluster3 (**C**), and cluster4 (**D**) in different time points.

**Figure 4:**
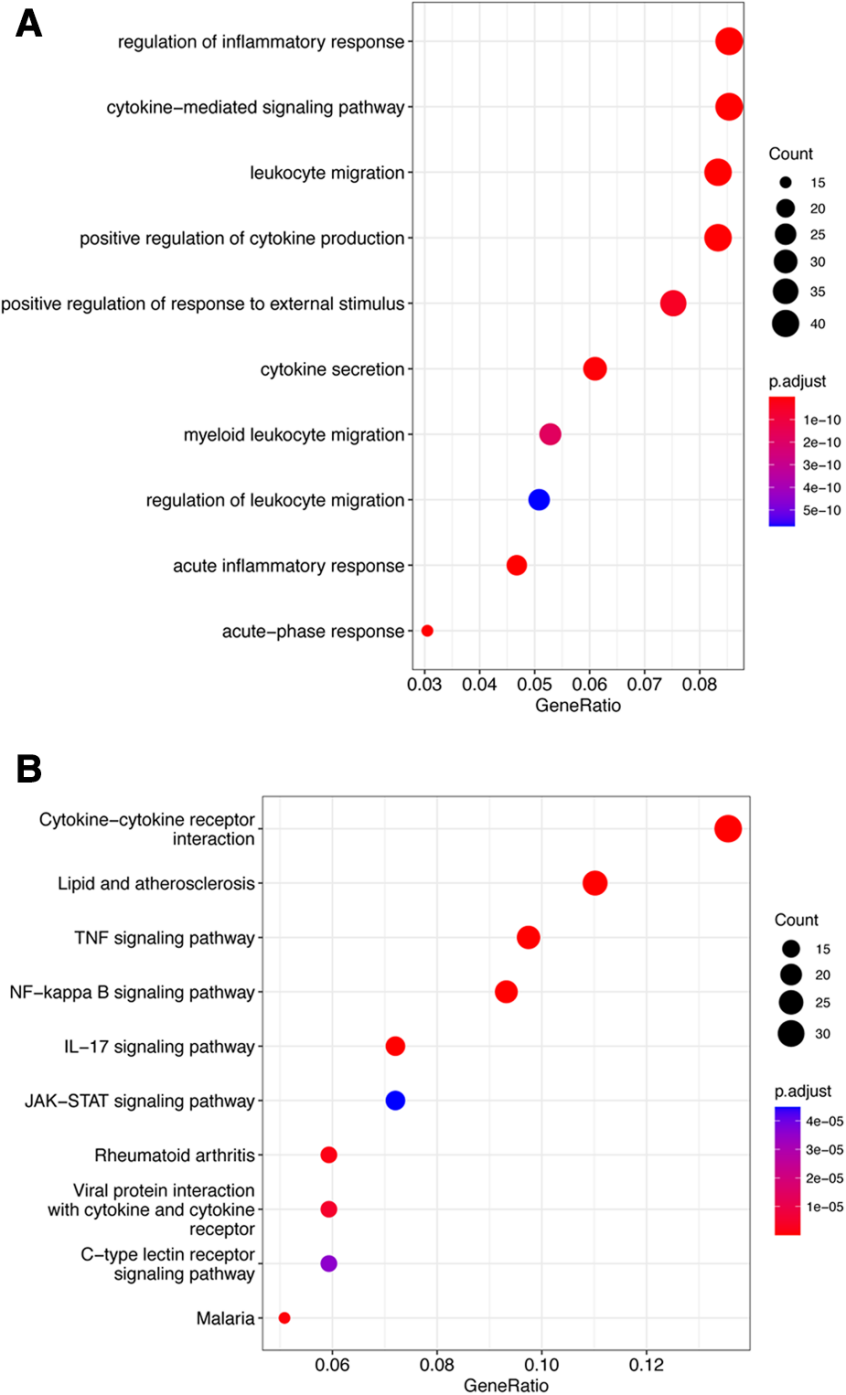
Functional enrichment analysis of cluster 4. (**A**) Top 10 terms in GO analysis of cluster 4 with *P* < 0.05. (**B**) Top 10 terms in the KEGG pathway analysis of cluster 4 *P* < 0.05. GO, Gene Ontology; KEGG, Kyoto Encyclopedia of Genes and Genomes.

The fuzzy c-means algorithm was then used to cluster gene expression profiles in all time points for GSE171546. In total, four distinct clusters of temporal patterns were obtained representing genes regulated differently (Figures 3A–D). The results showed that the trend of gene expression in cluster 4 was consistent with the trend of the Ferroptosis pathway *Z*-score (Figure 3D), indicating that the genes enriched in cluster 4 may have potential association with ferroptosis. In order to further investigate the functions of these genes in cluster 4, GO and KEGG enrichment analysis was performed. The results showed that genes in cluster 4 were enriched in GO terms such as a regulation of inflammatory response, a cytokine-mediated signaling pathway, and cytokine secretion (Figure 4A) and in KEGG terms such as cytokine–cytokine receptor interaction, a TNF signaling pathway, and a JAK-STAT signaling pathway (Figure 4B).

### 3.4. Identification of ferroptosis-associated targets (Hmox1 and Slc7a11)

**Figure 5:**
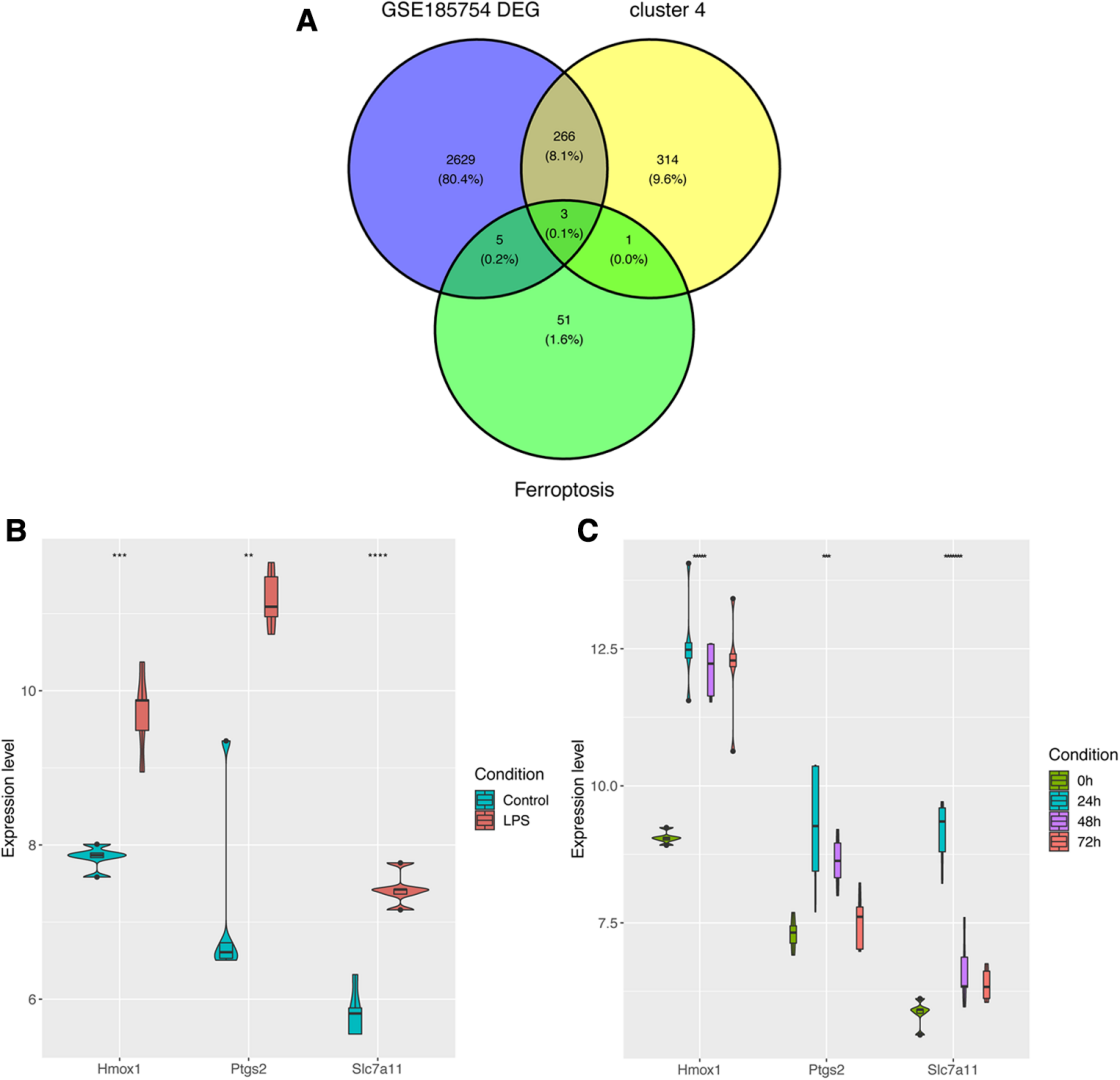
Identification of ferroptosis-associated targets. (**A**) Targets were selected through the overlap of DEGs, cluster 4, and FRGs. (**B**) The expression levels of Ptgs2, Hmox1, and Slc7a11 in GSE185754. (**C**) The expression levels of Ptgs2, Hmox1, and Slc7a11 in GSE171546. DEGs, differentially expressed genes; FRGs, ferroptosis-related genes; Ptgs2, prostaglandin endoperoxide synthase 2; Slc7a11, solute carrier family 7 member 11; Hmox1, heme oxygenase 1.

In order to further figure out ferroptosis-associated targets, all DEGs from GSE185754, cluster 4 from GSE171546, and the FRGs were intersected. A total of three targets were identified (Figure 5A). In addition to Ptgs2 as a recognized biomarker in sepsis-induced cardiac injury, Hmox1 and Slc7a11 were reported as ferroptosis-associated targets in sepsis cardiac dysfunction. Furthermore, the expressions of Hmox1 and Slc7a11 were also confirmed in GSE185754 and GSE171546. The results showed that Hmox1 and Slc7a11 expressed highly in the sepsis group than in the control group (Figure 5C). For the sake of comprehensiveness, GSE215955 and GSE53007 were used to validate the expression patterns of Hmox1 and Slc7a11, revealing that Hmox1 and Slc7a11 also had high expression in the sepsis group (Supplementary Figure S2).

### 3.5. Downregulation of ferroptosis-associated targets (Ptgs2, Hmox1, and Slc7a11) alleviated LPS-induced cardiac injury

**Figure 6:**
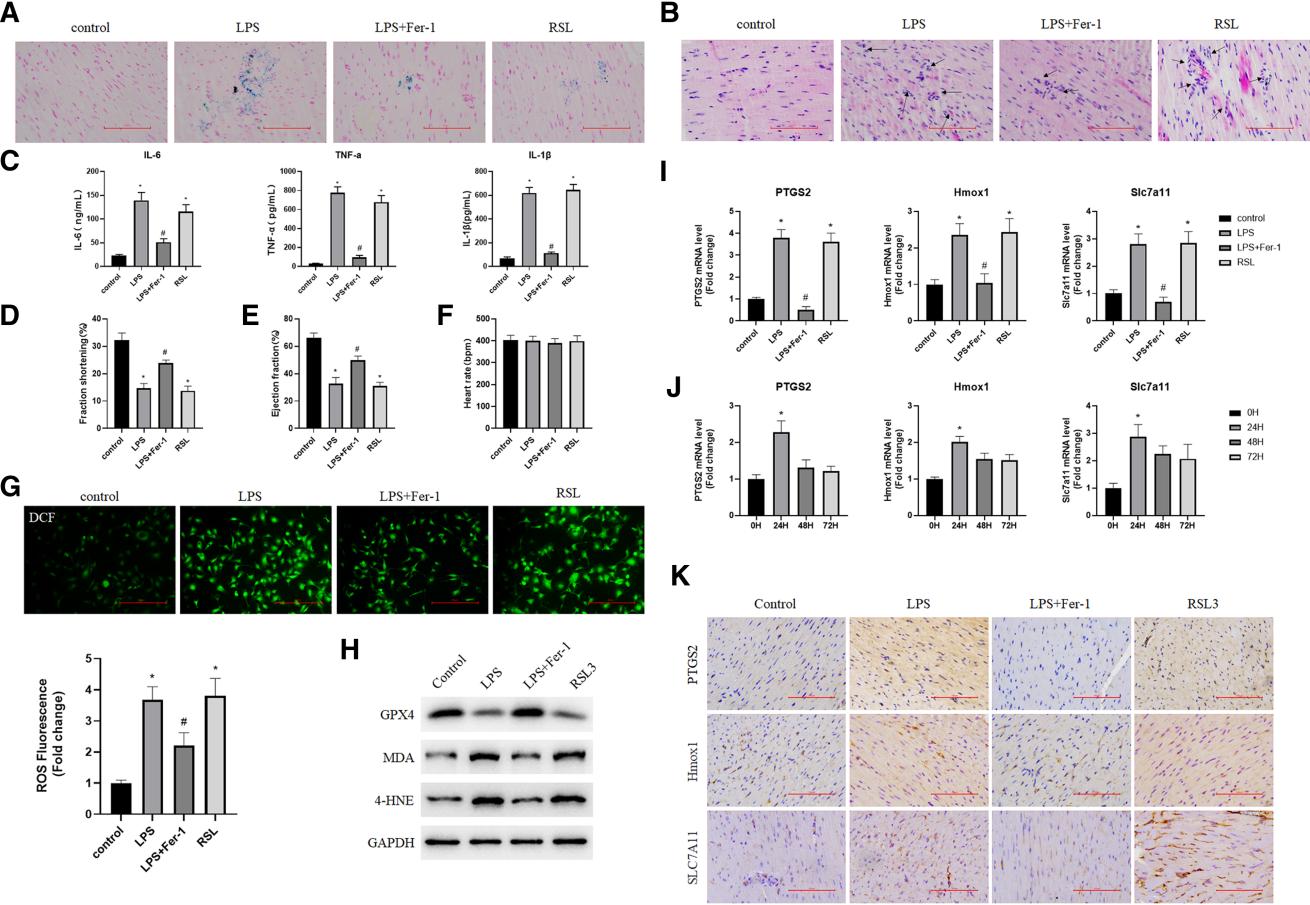
Validation of targets associated with iron poisoning in mice. (**A**) Iron staining of heart tissue in the normal control group, LPS group, LPS + Fer-1 group, and RSL3 group; (**B**) H&E staining of heart tissue in the normal control group, LPS group, LPS + Fer-1 group, and RSL3 groups (the arrows indicate iron-dead cells); (**C**) Serum inflammatory cytokines (IL-6, TNF-α, and IL-1β) were determined by ELISA. Compared with the control group, *P < 0.05, compared with the LPS group, #*P* < 0.05; (**D,E**) left ventricular shortening fraction and left ventricular ejection fraction in each group. Compared with the control group, *P < 0.05, compared with the LPS group, #*P* < 0.05; (**F**) heart rate of each group; (**G**) DCF-cellular ROS assay was used to assess the ROS level in the cardiac tissue samples quantitatively. (**H**) The expression levels of iron death marker protein (GPX4, MDA, and 4-HNE) were detected by using Western blot. (**I**) The expression of Ptgs2, Hmox1, and Slc7a11 genes in the heart tissue was detected by using qRT-PCR. Compared with the control group, *P < 0.05, compared with the LPS group, #*P* < 0.05; (**J**) Expression of Ptgs2, Hmox1, and Slc7a11 at 0, 24, 48, and 72 h after cardiac injury caused by sepsis. Compared with the 0 h group, *P < 0.05; (**K**) Representative immunohistochemical images of cardiac tissue Ptgs2, Hmox1 and Slc7a11. LPS, lipopolysaccharide; Fer-1, Ferrostatin-1; H&E, hematoxylin and eosin; ROS, reactive oxygen species; Ptgs2, prostaglandin endoperoxide synthase 2; Slc7a11, solute carrier family 7 member 11; Hmox1, heme oxygenase 1; DCF, Dichlorofluorescein.

To further validate the real expression of ferroptosis-associated targets, a model of sepsis-induced cardiac injury was established by an injection of LPS. The ELISA results showed that the serum levels of inflammatory cytokines IL-6, TNF-α, and IL-1β were significantly increased (Figure 6C), and iron and H&E staining also showed the successful construction of the model (Figures 6A,B). In addition, the WB results showed that the levels of iron death markers MDA and 4-HNE in the heart tissues from mice in the LPS and RSL3 groups were significantly increased, GPX4 levels were significantly reduced, and the levels of iron-mediated cell death were significantly increased. However, compared with the LPS group, the LPS + Fer-1 group showed a significant decrease in iron-mediated cell death (Figure 6H). In addition, pretreatment with Fer-1 elevated fraction shortening % and ejection fraction % in LPS-treated mice (Figures 6D–E), while the heart rate of mice in all groups exhibited no difference (Figures 6F). Accumulation of ROS is one of the most important pathological features of septic cardiomyopathy. We further examined the ROS level in each group and the results showed that the expression of ROS in the cardiac tissues of the LPS + Fer-1 group was significantly lower than that of the LPS group (Figure 6G). All the above results showed that inhibition of ferroptosis could reverse the cardiac dysfunction in sepsis and revealed the successful construction of mouse models. In order to further investigate the role of Ptgs2, Hmox1, and Slc7a11 in sepsis-induced cardiac injury, we first investigated their expression levels in each group. Compared with the control group, the fold change of Ptgs2, Hmox1, and Slc7a11 confirmed that the expression of ferroptosis-associated targets was higher in the LPS group and can be decreased by Fer-1 (Figure 6I). In addition, the time-course expressions of Ptgs2, Hmox1, and Slc7a11 in the model of sepsis-induced cardiac injury induced by the injection of LPS were also analyzed. The fold change of Ptgs2, Hmox1, and Slc7a11 in sepsis-induced heart injury increased in 24 h and decreased in 48 and 72 h, which exhibited the same time trend as found previously (Figures 6J,K).

**Figure 7:**
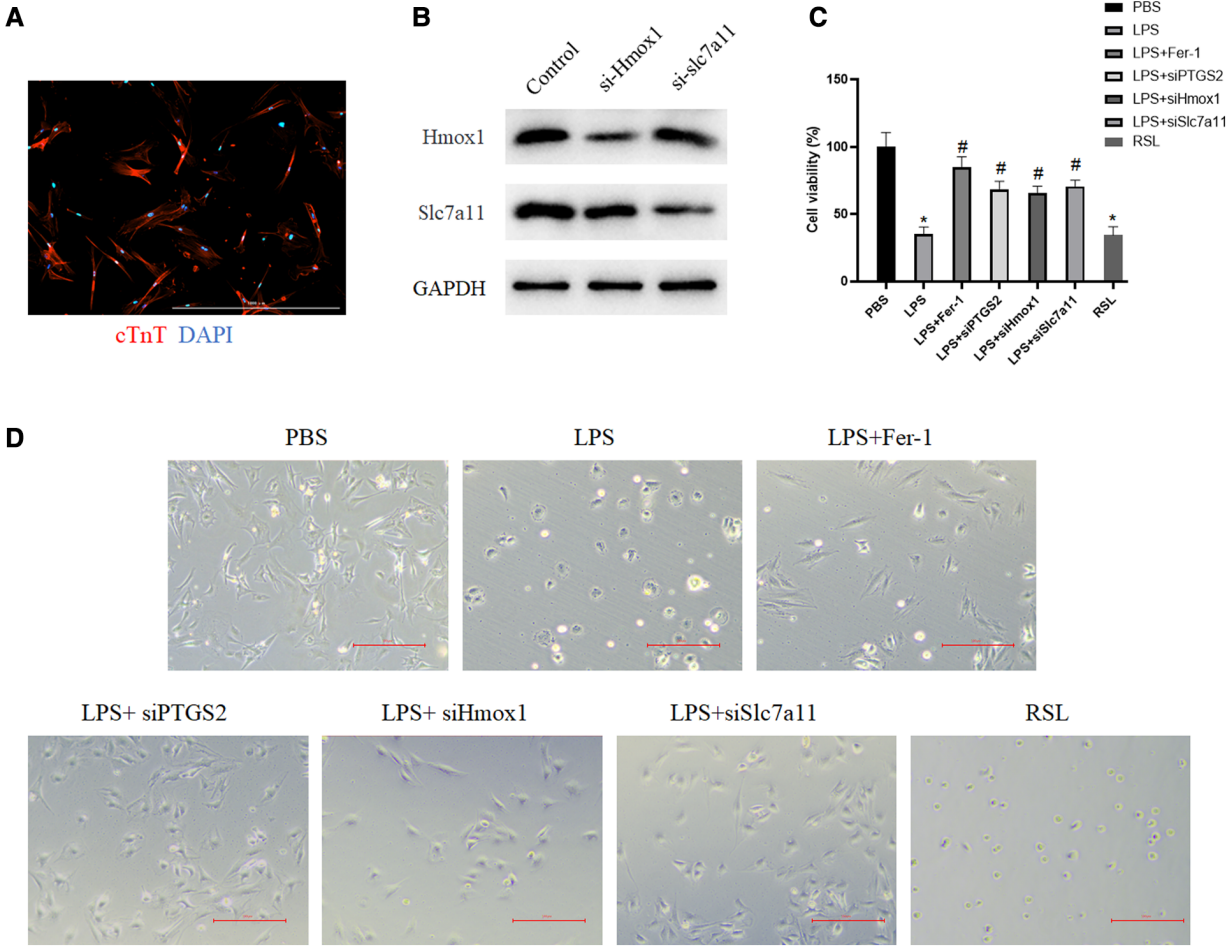
Downregulation of these identified targets increased myocardial cell viability. (**A**) Primary cardiomyocytes were immunostained with cardiac troponin T (red) and the nuclei were stained with DAPI (blue); (**B**) Western blot verified the downregulation of Hmox1 and slc7a11. (**C**) The viability of myocardial cells after the downregulation of Ptgs2, Hmox1, and slc7a11 was measured by using CCK-8. The asterisk “*” indicates a statistically significant comparison with the PBS (Phosphate Buffered Saline) group. “#” indicates no significant difference with the PBS group. (**D**) Morphology of myocardial cells in the PBS group, LPS group, LPS + si-Ptgs2 group, LPS + si-Hmox1 group, LPS + si-Slc7a11 group, and LPS + Fer-1 group under light microscope. Ptgs2, prostaglandin endoperoxide synthase 2; Slc7a11, solute carrier family 7 member 11; Hmox1, heme oxygenase 1; LPS, lipopolysaccharide.

Although the expressions of Ptgs2, Hmox1, and Slc7a11have been confirmed in two datasets, the functions of these ferroptosis-related targets remained unknown. The rat myocardial primary cells were then obtained (Figure 7A) and transfected with si-Hmox1 and si-Slc7a11. Western blot analysis was performed to assess the knockdown efficiency (Figure 7B). The results of CCK-8 showed that after downregulation of these targets, the viability of myocardial cells treated with LPS was increased significantly compared with the LPS group, which exhibited the same function of Fer-1 (Figure 7C). Downregulating the expression of Ptgs2, Hmox1, and Slc7a11 resulted in the restoration of cardiac cell morphology from swelling and cell death to a normal state (Figure 7D).

## 4. Discussion

Sepsis is usually defined as a life-threatening infection associated with organ dysfunction (18). It has a potentially complicated mechanism (19). A groundbreaking study has provided comprehensive insights into the intricate mechanisms underlying sepsis-induced cardiac injury. These mechanisms involve uncontrolled inflammation and accumulation of oxidative stress primarily created by impaired host response to infection (1). In theory, a series of strategies have been demonstrated for treating sepsis-induced cardiac injury and have been considered highly appealing. However, all these treatments are clinically less effective (20). In recent years, researchers have reported that ferroptosis may be one of the potential mechanisms of heart injury caused by sepsis (7). As proposed by Professor Brent first in 2012, iron-dependent accumulation of lipid peroxidation generally occurs during ferroptosis (9). In the process of ferroptosis, mitochondria undergo changes such as reduction or loss of mitochondrial ridges, increased density of the mitochondrial membrane, and rupture of the outer membrane (21). In our study, a comprehensive and detailed investigation of ferroptosis in sepsis-induced cardiac injury was performed based on mfuzz analysis and other bioinformatic strategies based on GSE185754 and GSE171546 datasets to identify novel targets.

After primary screening, 2903 DEGs were selected from GSE185754 and 1945 DEGs were selected from GSE171546. As revealed by the functional enrichment analysis, genes of the sepsis group were more likely to be enriched in the ferroptosis pathway than those of the control group from both datasets, which was consistent with the findings of previous research demonstrating that ferroptosis is closely related to heart injury (7). Interestingly, the ferroptosis pathway Z-score increased rapidly in the first 24 h after CLP, reached a peak in 24 h, and then gradually decreased from 24 to 72 h. This observation indicated that ferroptosis physiological processes may be concentrated on the first 24 h and then decrease. To gain further insights into the role of ferroptosis in sepsis-induced cardiac injury, the fuzzy c-means algorithm was used to cluster gene expression profiles at different time points such as 0-, 24-, 48-, and 72-h points. Cluster 4 was then found to be consistent with the ferroptosis pathway *Z*-score in the CLP models for the same trend. After an intersection of DEGs in GSE185754, cluster 4, and ferroptosis-related genes, a total of three targets was finally determined: Ptgs2, Hmox1, and Slc7a11.

As a well-recognized marker of ferroptosis (22), Ptgs2, also known as cyclooxygenase-2 (COX-2), has shown a high level of expression in sepsis-induced cardiac injury (23, 24). As the most important rate-determining enzyme in arachidonic acid metabolism, Ptgs2 is closely associated with inflammation and ferroptosis (25). Previous research has demonstrated that cardiomyocytes, when exposed to LPS stimulation, exhibit an upregulation in the expression of Ptgs2, and pretreatment with Fer-1 or DXZ revealed a reduction in both Ptgs2 levels and lipid ROS in cardiomyocytes, both in *in vivo* and *in vitro* settings (23, 24). Our study showed the same phenomenon that Ptgs2 expressed highly in the LPS group. Meanwhile, two novel ferroptosis-related targets were also selected in sepsis-induced cardiac injury: Hmox1 and Slc7a11.

In recent times, Hmox1 has drawn significant attention of scientists as an important ferroptosis-associated biomarker in many diseases, which can be used to regulate ferroptosis for disease treatment (26–29). It was reported that inhibiting the expression of Hmox1 in diabetic human endothelial cells can attenuate Fe^2+^ overload, which further reduces ROS and iron content, and alleviates lipid peroxidation, indicating the vital role of Hmox1 in ferroptosis (27). It was also shown that cephalosporin antibiotics induced Hmox1 specifically and selectively, which finally activated ferroptosis and inhibited the proliferation of nasopharyngeal carcinoma (28). Interestingly, Hmox1 has also been widely regarded as a potent cardioprotective target. Overexpression of Hmox1 was found to alleviate cardiac ischemia/reperfusion injury and protect persistent heart failure from coronary ligation (30, 31). However, transgenic mice that overexpressed Hmox1 were found to rapidly develop spontaneous heart failure within 1 year (32). Also, our study showed that the high expression of Hmox1 had a close relationship with sepsis-induced cardiac injury. These results indicated that Hmox1 played a pathogenic role in sepsis-induced cardiac injury.

Slc7a11 encoded multichannel transmembrane protein that mediated cystine/glutamate antiporter activity in the Xc system (33). As a ferroptosis-associated biomarker, Slc7a11 has been investigated in many diseases, especially in cancer and its treatment. Recently, a growing number of studies have highlighted the role of Slc7a11 overexpression in partially promoting tumor growth by inhibiting ferroptosis, suggesting that Slc7a11 exhibited antiferroptosis function during malignant progression (34–36). The potential mechanism was that SLC7A11 inhibits ferroptosis by facilitating the import of cystine, which subsequently enhances the biosynthesis of glutathione and facilitates GPX4-mediated detoxification of lipid peroxides (37). Earlier investigations have also demonstrated that the direct inhibition of SLC7A11 transporter activity by certain drugs can cause ferroptosis and inhibit tumor growth *in vivo* (38, 39). However, not all elevated Slc7a11 expression is associated with the presumed function of reducing ferroptosis, especially in inflammatory models (40–42). Hui’s group reported that the decrease of Slc7a11 expression markedly increased the expression of Nrf2-HO-1 and then cell death in reoxygenation (OGD/R) and oxygen-glucose deprivation models attenuated dramatically (36). The potential mechanisms may contribute to the double-edged sword feature of Slc7a11 as in regulating nutrient dependency and the redox balance (42). The findings from our study revealed elevated levels of Slc7a11 in the sepsis group, indicating that the more complicated mechanisms between Slc7a11 and ferroptosis in various diseases should be investigated in the future.

After identifying two novel targets in sepsis-induced cardiac injury through bioinformatic methods, we further validated their expression and functions *in vivo* and *in vitro*. It has been shown that Ptgs2, Hmox1, and Slc7a11 were highly expressed in the LPS group and can be inverted by Fer-1. Also, rat myocardial primary cells were transfected with siRNA to decrease the expression of Ptgs2, Hmox1, and Slc7a11. By analyzing cell viability and cell morphology, the downregulation of Ptgs2, Hmox1, and Slc7a11 reversed the weak status of cells caused by LPS, which exhibited the same antiferroptosis of Fer-1. Although both bioinformatic and experimental evidence has shown that Ptgs2, Hmox1, and Slc7a11 had the potential to be considered ferroptosis-associated targets in sepsis-induced cardiac injury, the question remains whether Hmox1 and Slc7a11 were just general targets of general inflammation in cardiac injury. In fact, numerous studies have consistently highlighted the significant role of ferroptosis in the development of sepsis-induced heart injury (7), and Ptgs2, Hmox1, and Slc7a11 are well-recognized ferroptosis-associated targets by academia (22, 26, 37). Meanwhile, our findings indicate a clear correlation between the gene expression patterns of Ptgs2, Hmox1, and Slc7a11 and the changes in the Ferroptosis pathway Z-score during sepsis-induced heart injury (Figures 2D, 5C), indicating the specific relationship among Ptgs2, Hmox1, Slc7a11, and ferroptosis in sepsis-induced heart injury. In addition, as we mentioned previously, the expressions of Hmox1 and Slc7a11 were also evaluated in some inflammatory diseases of the myocardium. No clear detectable differences were observed in myocardial infarction and myocarditis (Supplementary Figure S3). However, due to the complicated relationship between ferroptosis and inflammation, it is hard to demonstrate that there is no effect of general inflammation in heart injury involved in the regulation of Hmox1and Slc7a11. Additional investigations are possibly required to investigate the underlying mechanisms of action in greater detail.

Therefore, our study first demonstrated two novel ferroptosis-associated targets in sepsis-induced cardiac injury: Hmox1 and Slc7a11 through bioinformatic selection and laboratory measurement. These two identified targets could be regarded as potential therapeutic and diagnostic targets for sepsis-induced cardiac injury. However, due to the limited datasets with no clinical validation, there is still room for further investigation.

## 5. Conclusion and limitations

In conclusion, all selected DEGs were mainly enriched in ferroptosis-related GO terms and KEGG pathways. The ferroptosis pathway was found to rapidly increase in the first 24 h, reaching a peak in 24 h, and decreasing gradually in the 24–72 h. A total of three ferroptosis-related targets (Ptgs2, Hmox1, and Slc7a11) were finally identified and found to be expressed highly in sepsis-induced cardiac injury. The role of Ptgs2 in sepsis has been well established. Therefore, this study does not elaborate on it. Hmox1 and Slc7a11 were reported to be involved in the progression of sepsis-induced cardiac injury. These two novel targets exhibited high expression in mice with LPS-induced heart dysfunction, and the viability of LPS-treated myocardial cells can be reversed by a downregulation of Hmox1 and Slc7a11. Our findings may help find targets for the treatment of sepsis-induced cardiac injury and improve existing therapies for patients with sepsis.

Despite the encouraging results of this study, some limitations remain. First, our study shows a high expression of Slc7a11 in the sepsis group, suggesting that the investigation of a more possible complex mechanism between Slc7a11 and ferroptosis in sepsis is required. Second, due to the complex relationship between ferroptosis and inflammation, it is difficult to confirm that Hmox1 and Slc7a11 are not involved in general inflammation in heart injury, and their mechanisms of action deserve to be further investigated. Finally, the results of this study have not been clinically verified in patients, and therefore, its clinical effect remains to be further confirmed.

## Data Availability

Publicly available datasets were analyzed in this study. These datasets can be found here: Gene Expression Omnibus (GEO) (http://www.ncbi.nlm.nih.gov/geo/), accession numbers: GSE185754, GSE171546, GSE215955, and GSE53007.

## Supplementary material

SUPPLEMENTARY FIGURE S1

The distribution of read counts in GSE185754 and GSE171546 before and after normalization. The original sequencing files were obtained following the instructions of the respective datasets of GSE websites and the R language “Seq2” package (R version 4.1.2) was used to perform differential expression analysis. The distribution of read counts in GSE185754 was shown before (Supplementary figure 1A) and after (Supplementary figure 1C) normalization. The distribution of read counts in GSE171546 was also shown before (Supplementary figure 1B) and after (Supplementary figure 1D) normalization.

SUPPLEMENTARY FIGURE S2

The validation of 3 ferroptosis-associated targets in GSE215955 and GSE53007. For the sake of comprehensiveness, GSE53007 and GSE215955 were also used to validate the expression pattern of Ptgs2, Hmox1 and Slc7a11. In GSE53007, RNA from murine heart muscle tissue (n=4 for sepsis, n=4 for control) was extracted and it demonstrated that the expression of Hmox1, and Slc7a11 was significantly higher in sepsis-induced heart injury group (Supplementary figure 2A). Due to the limitations of GSE53007 that didn”t include Ptgs2, the expression of Ptgs2 cannot be performed in GSE53007. In GSE215955, RNA sequencing was performed in heart of C57BL/6 mice from control and LPS-induced heart injury group (n=4 for each group). The result showed that the expression of Ptgs2, Hmox1, and Slc7a11 was significantly higher in sepsis-induced heart injury (Supplementary figure 2B).

SUPPLEMENTARY FIGURE S3

The expression of Hmox1 and Slc7a11 in inflammatory diseases of the myocardium. In order to further clarify that Hmox1 and Slc7a11 are ferroptosis-associated specific targets in sepsis-induced heart injury rather than general targets consequence of cardiac injury, we validated the expression of Hmox1 and Slc7a11 in myocarditis and myocardial infarction (MI). The datasets GSE53607 was used as myocarditis model that included 5 Theiler”s murine encephalomyelitis virus (TMEV)- infected mice and 5 control mice. The datasets GSE66360 was used as myocardial infarction model that included 20 patients with acute myocardial infarction and 9 healthy cohorts. The results showed that there was no differential expression of Hmox1and Slc7a11 in myocarditis (Supplementary figure 3A-B) and myocardial infarction (Supplementary figure 3C-D).
