# Supplementary figures and images for: Identification of two novel ferroptosis-associated targets in sepsis-induced cardiac injury: Hmox1 and Slc7a11
Source: Front Cardiovasc Med. 2023 Jun 23;10:1185924. doi: 10.3389/fcvm.2023.1185924 (PMC10326630; doi:10.3389/fcvm.2023.1185924)

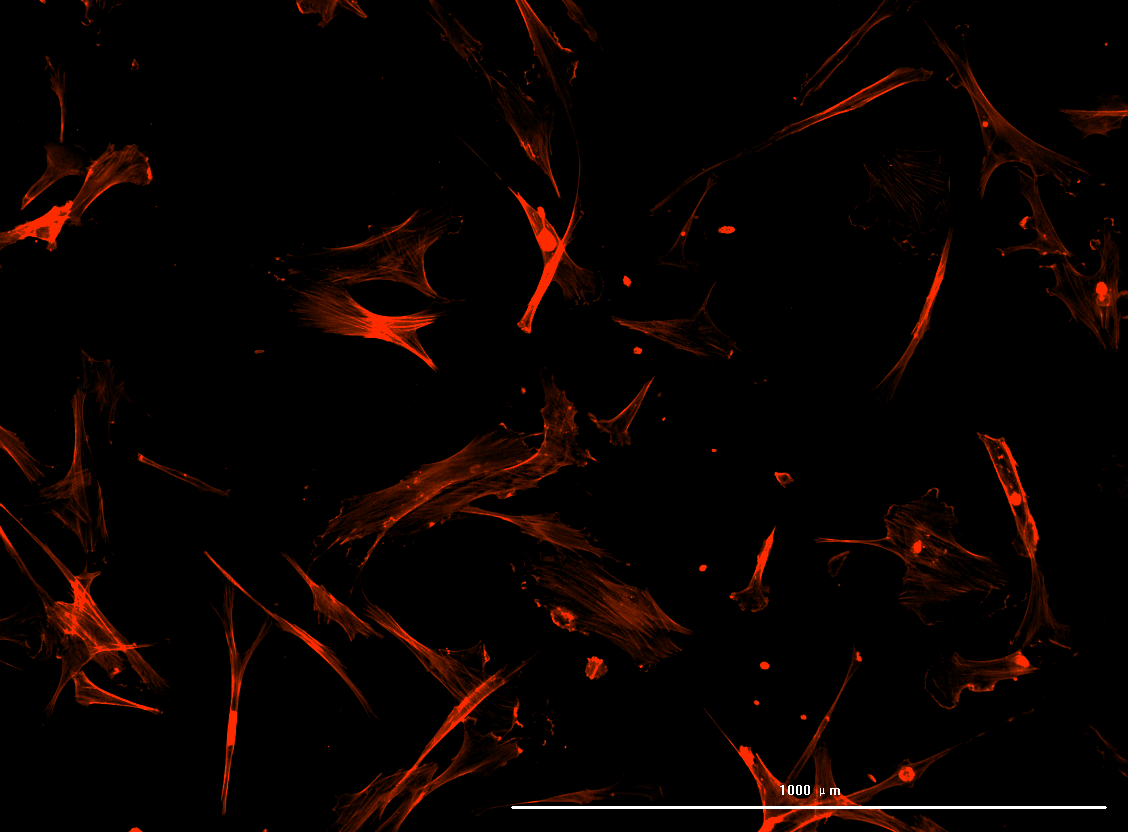

Supplement: Supplementary file 2 [file Datasheet2.zip › raw data for Figure 7/Figure 7A-Cell immunofluorescence/cTnT.png]

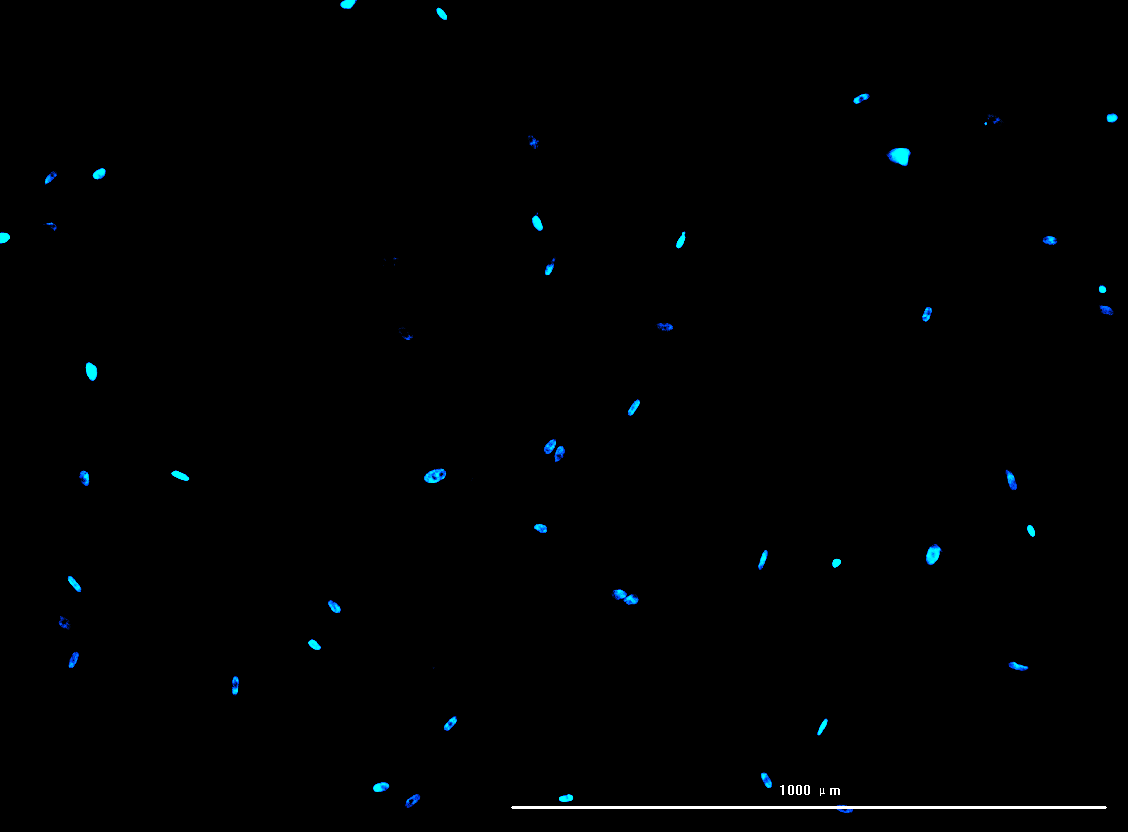

Supplement: Supplementary file 2 [file Datasheet2.zip › raw data for Figure 7/Figure 7A-Cell immunofluorescence/DAPI.png]

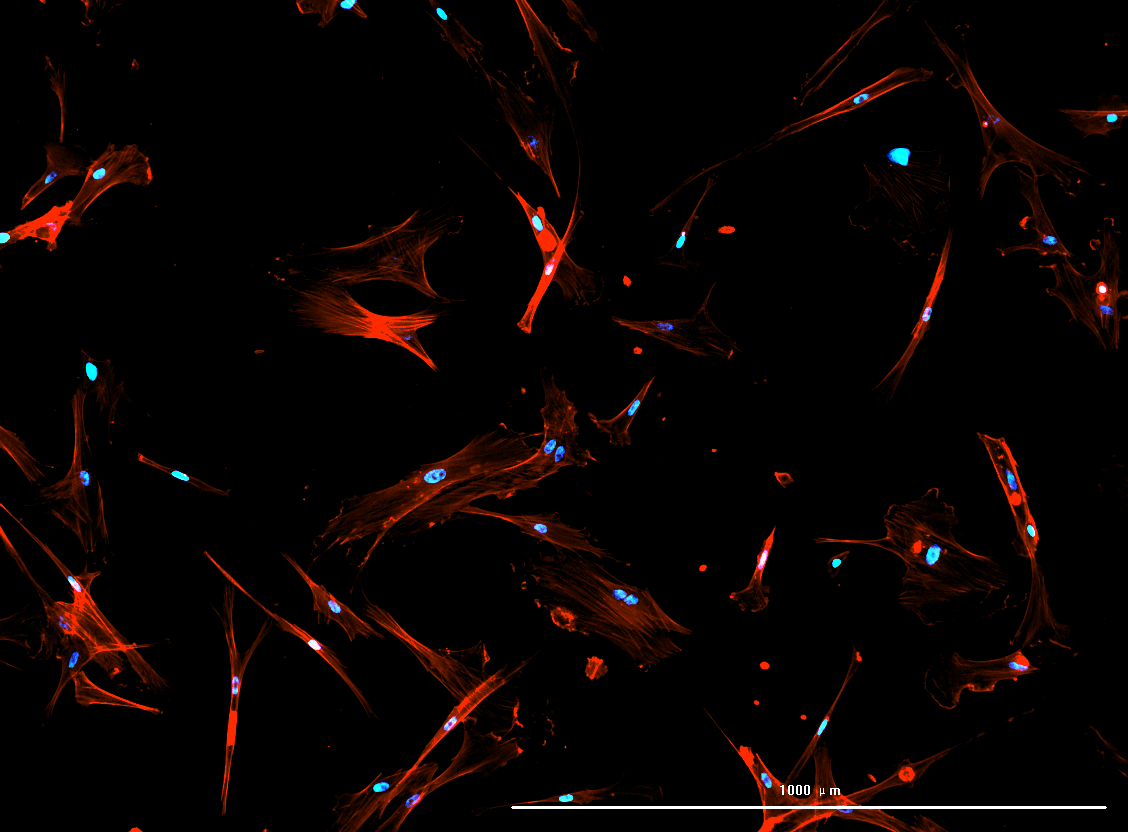

Supplement: Supplementary file 2 [file Datasheet2.zip › raw data for Figure 7/Figure 7A-Cell immunofluorescence/Merge.png]

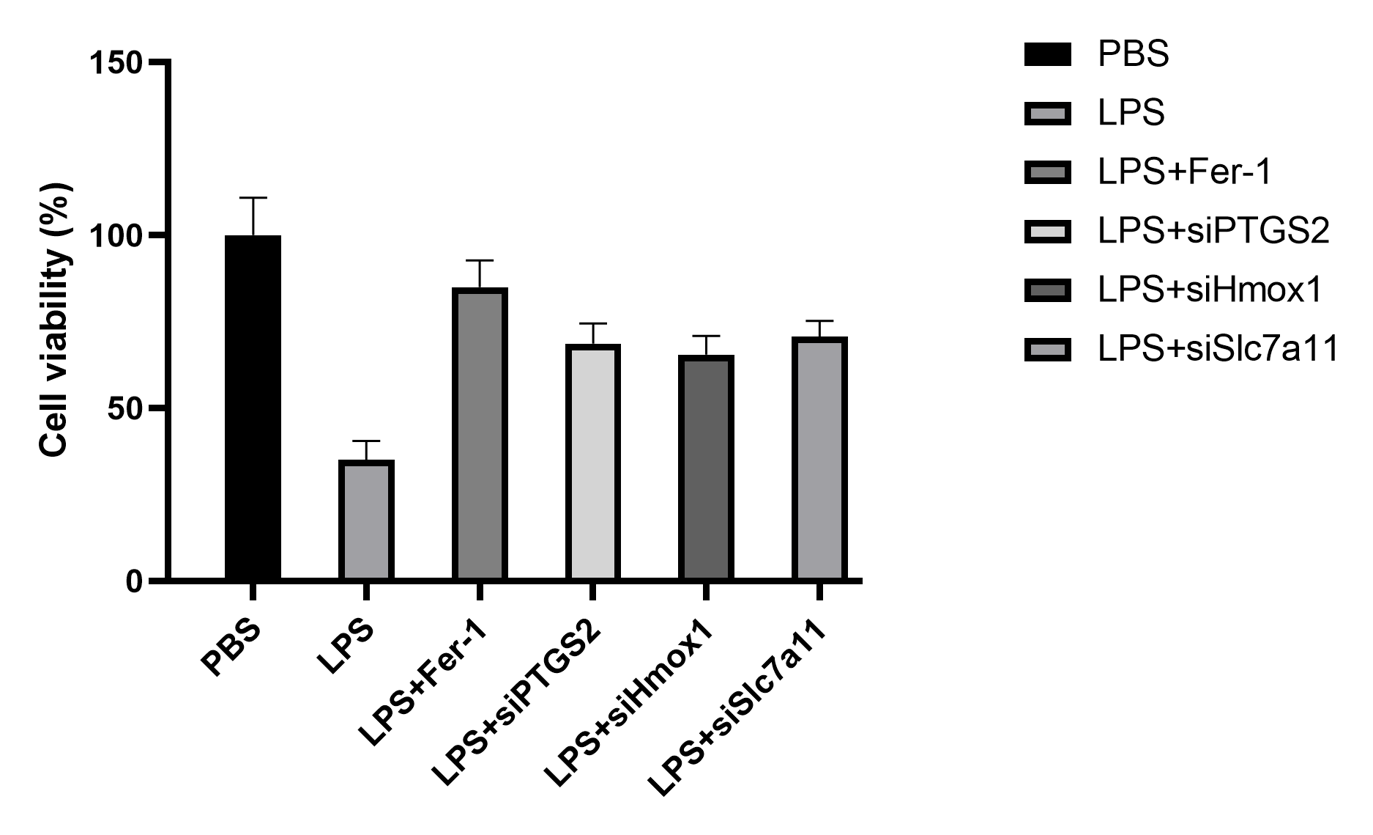

Supplement: Supplementary file 2 [file Datasheet2.zip › raw data for Figure 7/Figure 7B/Cell viability.tif]

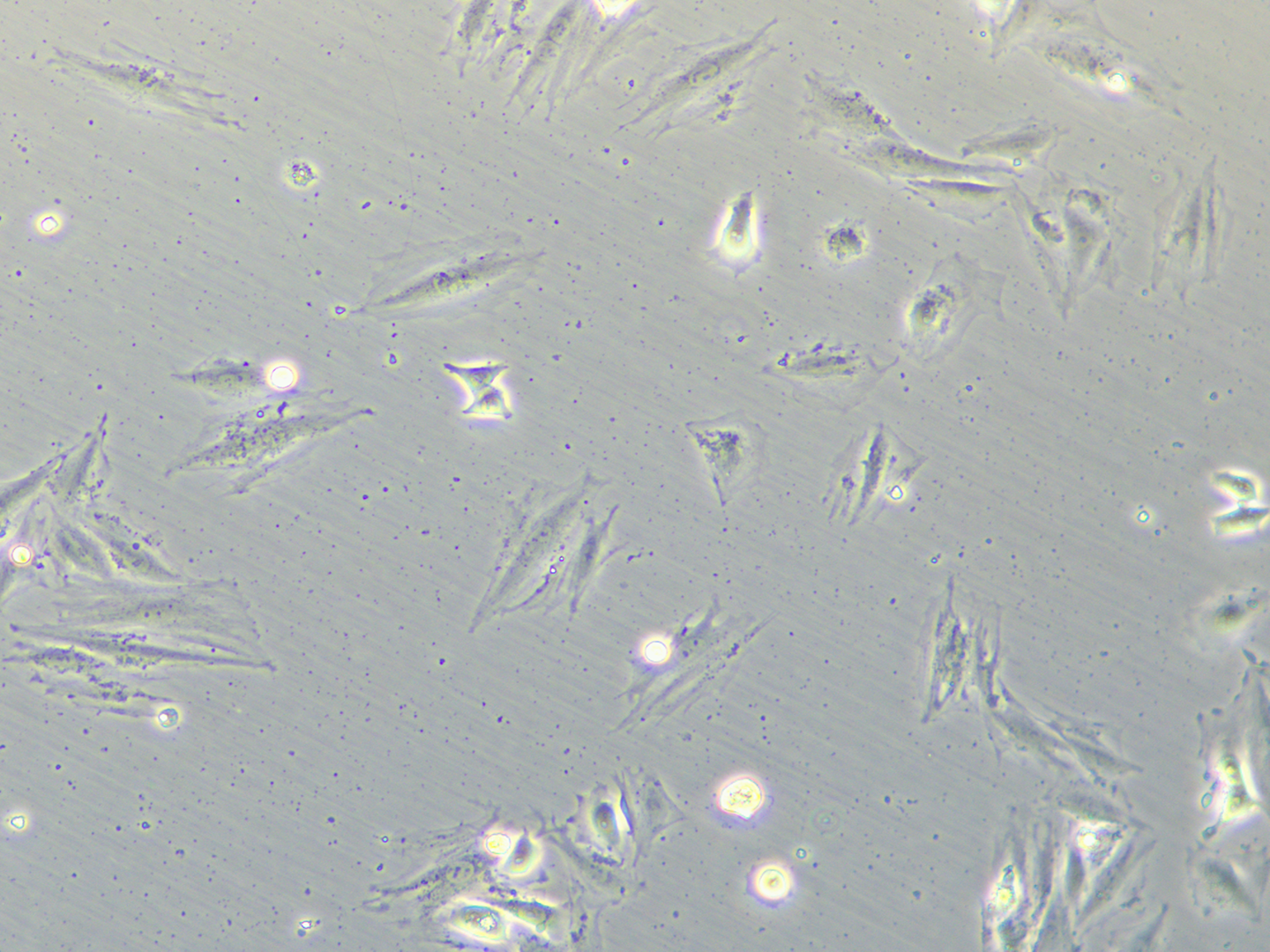

Supplement: Supplementary file 2 [file Datasheet2.zip › raw data for Figure 7/Figure 7C/LPS+Fer-1.tif]

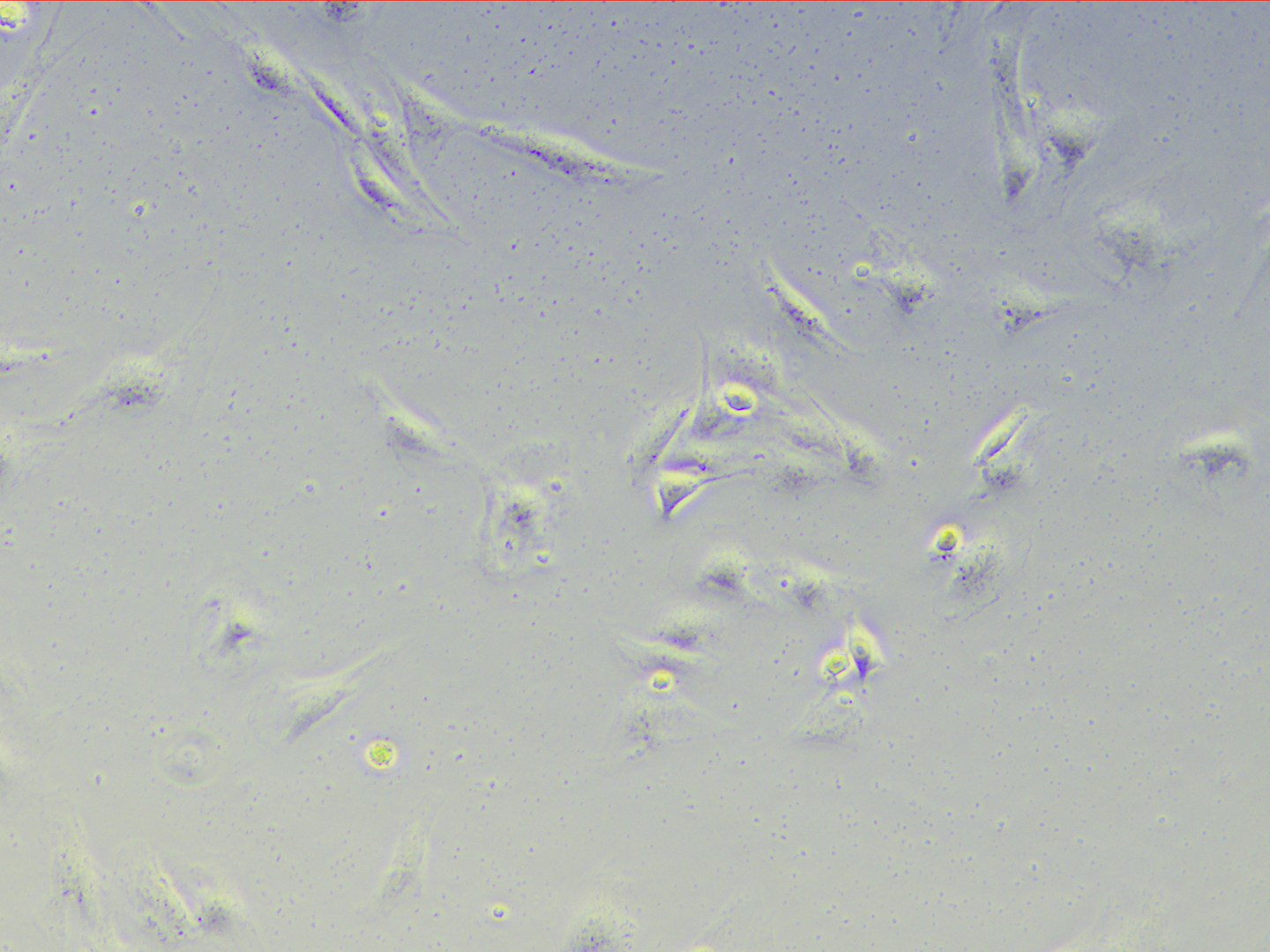

Supplement: Supplementary file 2 [file Datasheet2.zip › raw data for Figure 7/Figure 7C/LPS+siHomx1.tif]

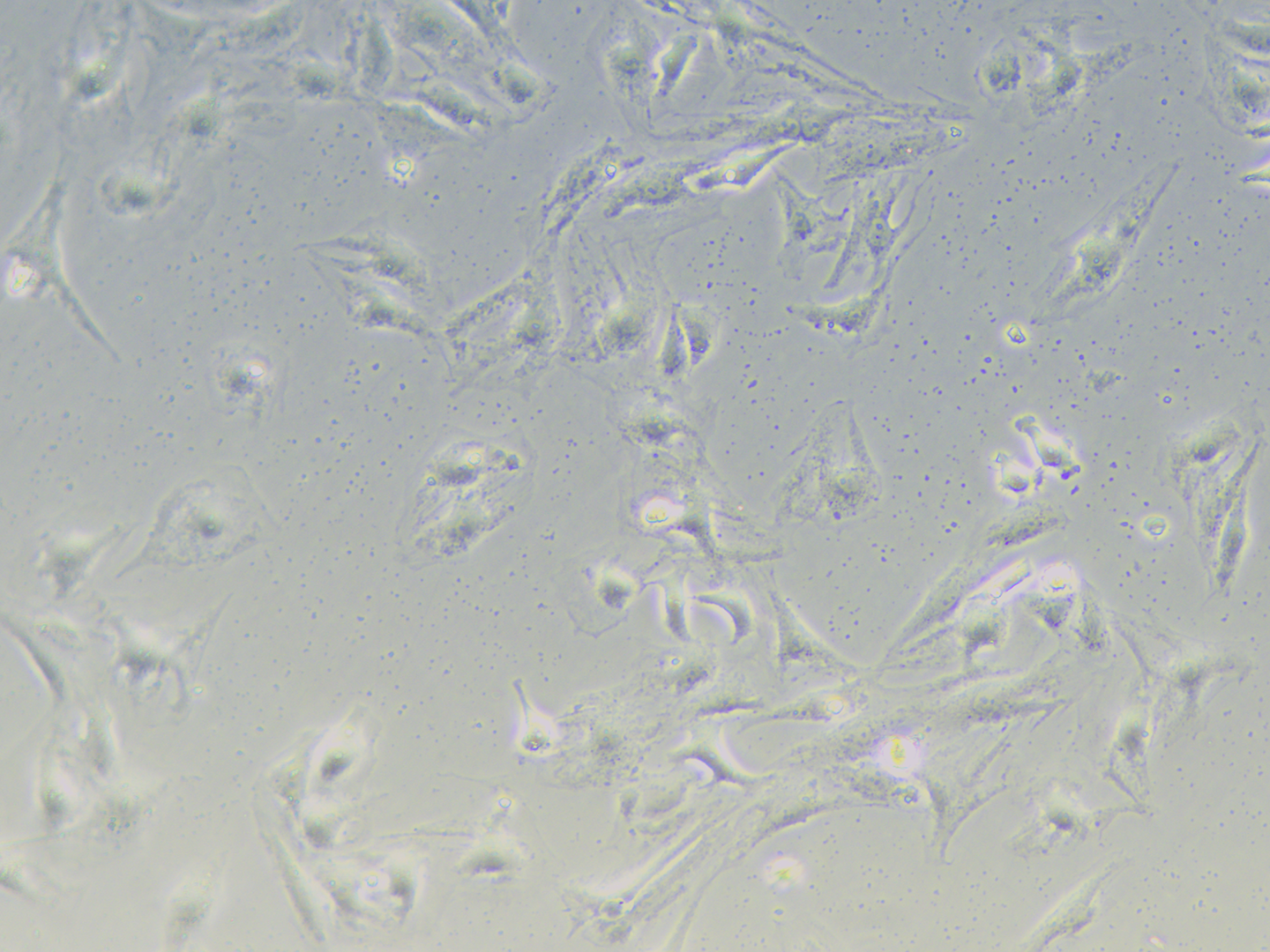

Supplement: Supplementary file 2 [file Datasheet2.zip › raw data for Figure 7/Figure 7C/LPS+siPTGS2.tif]

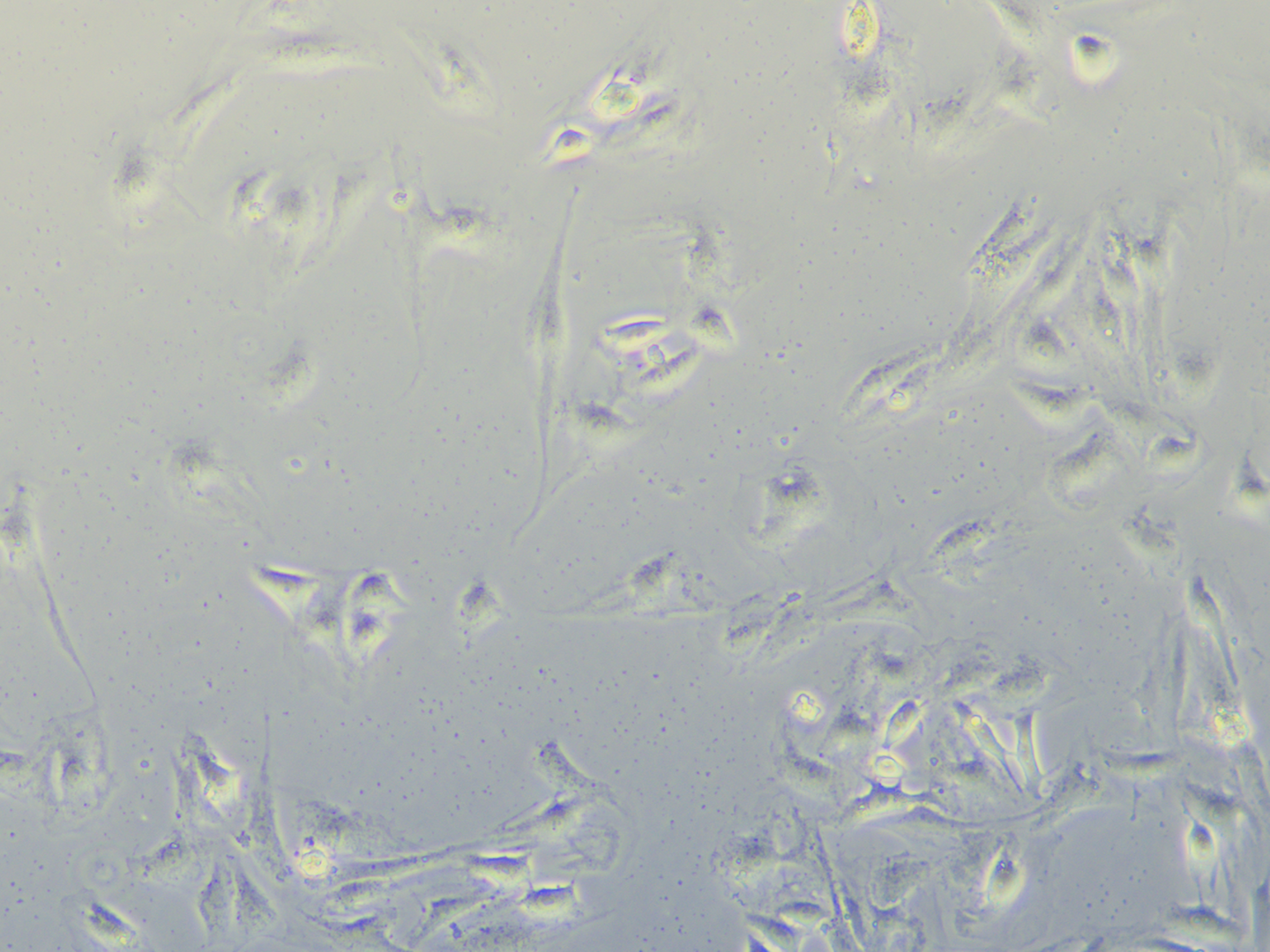

Supplement: Supplementary file 2 [file Datasheet2.zip › raw data for Figure 7/Figure 7C/LPS+siSlc7a11.tif]

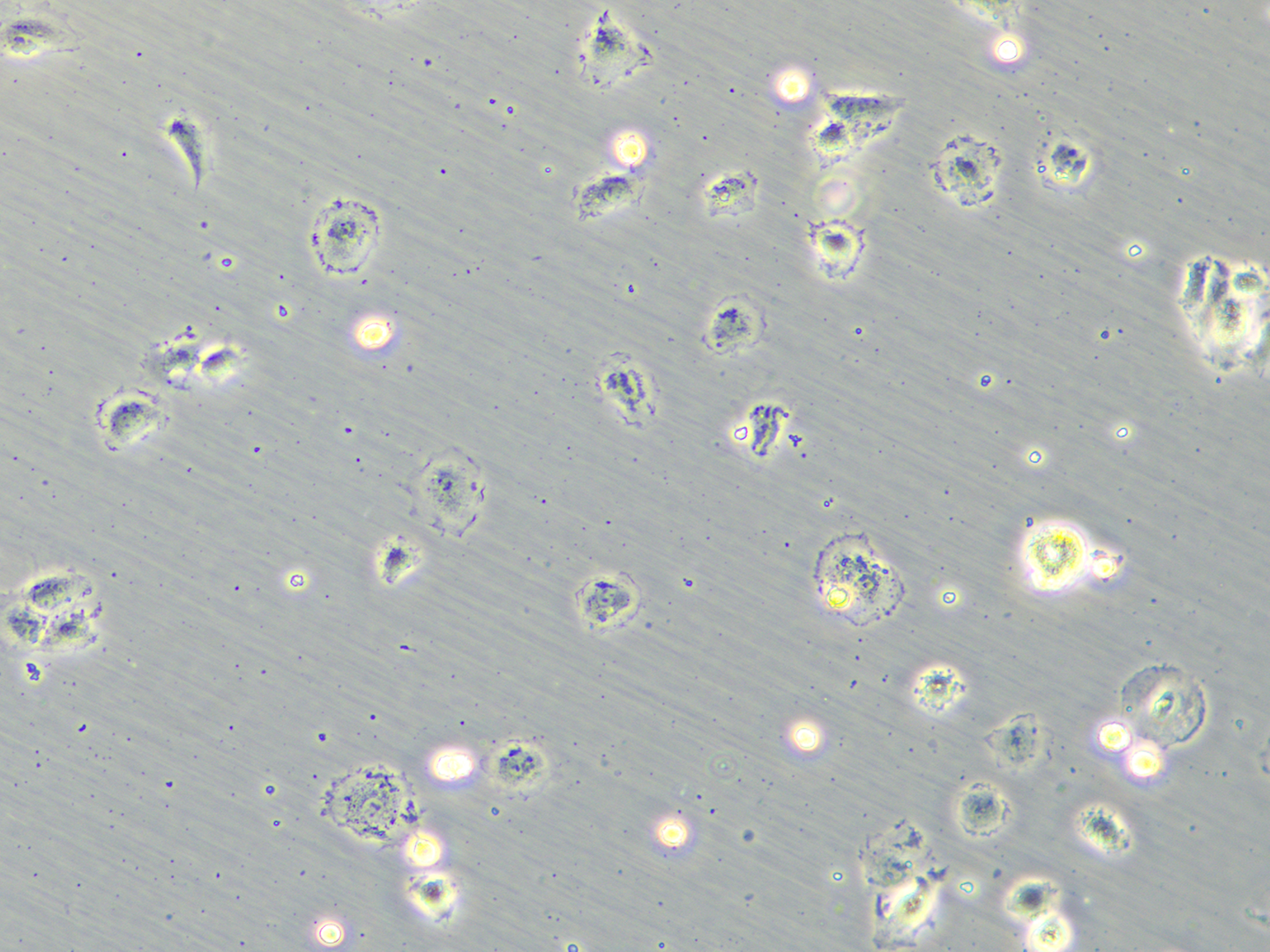

Supplement: Supplementary file 2 [file Datasheet2.zip › raw data for Figure 7/Figure 7C/LPS.tif]

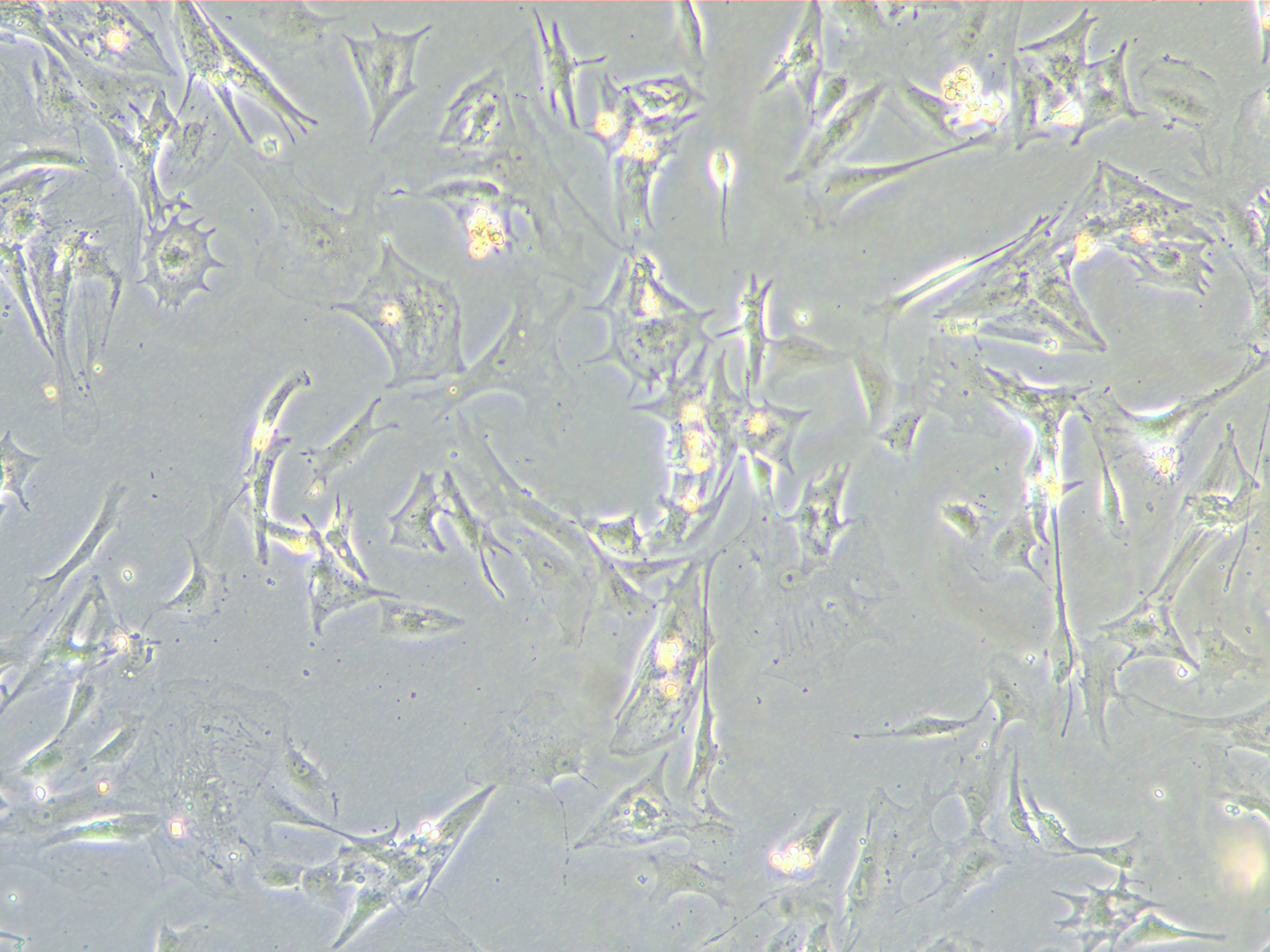

Supplement: Supplementary file 2 [file Datasheet2.zip › raw data for Figure 7/Figure 7C/PBS.tif]

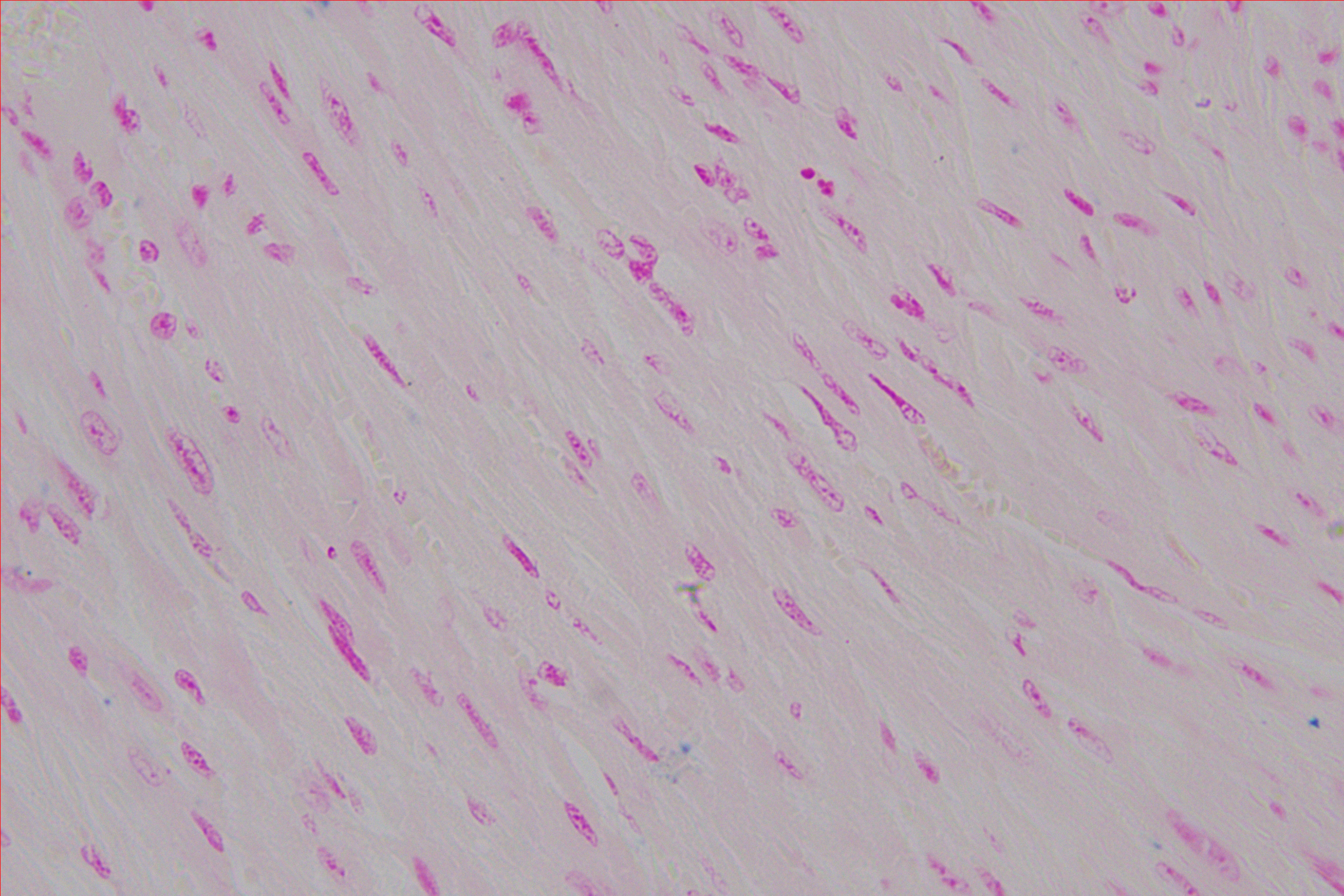

Supplement: Supplementary file 3 [file Datasheet3.zip › raw data for Figure 6/Figure 6 A/Control.jpg]

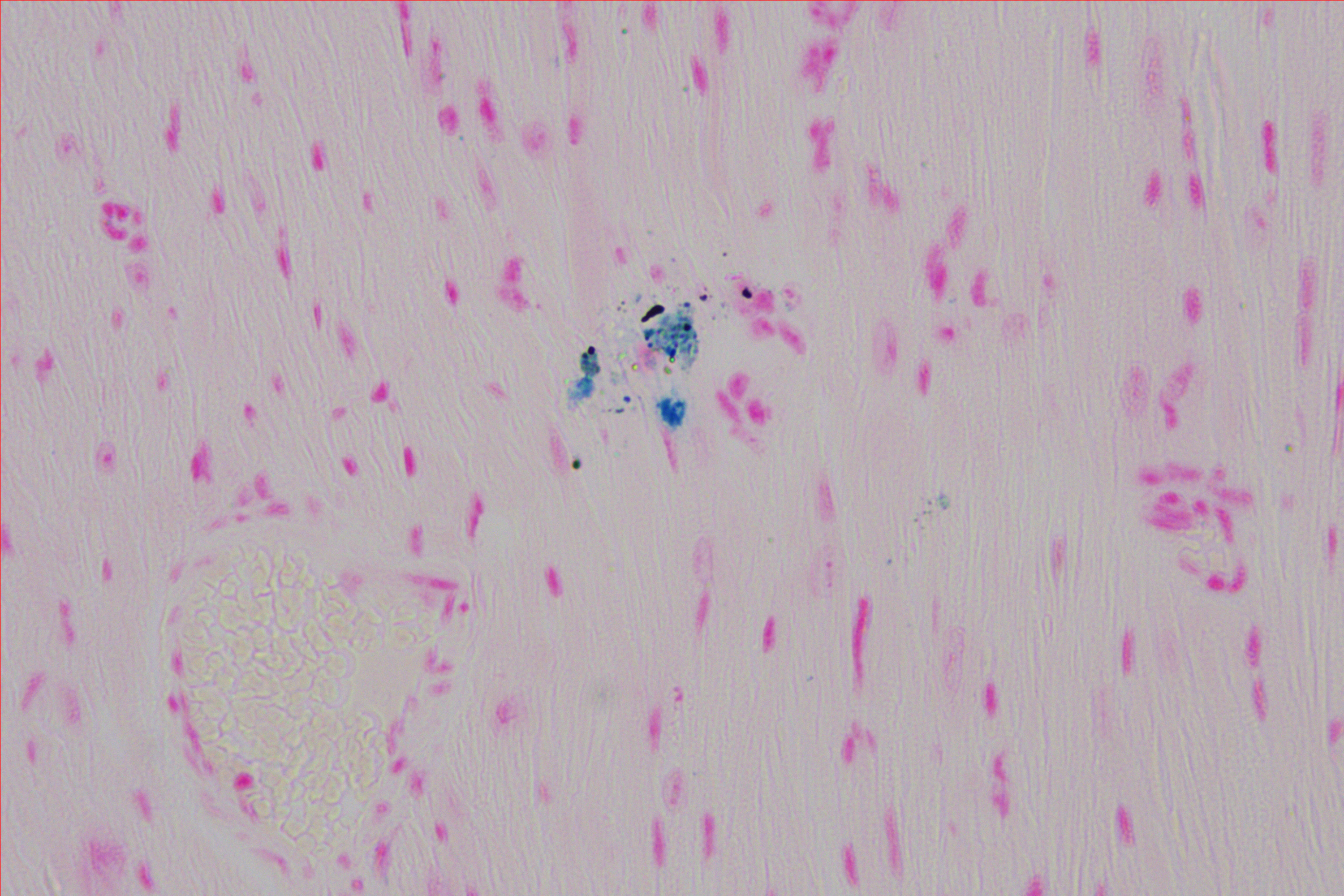

Supplement: Supplementary file 3 [file Datasheet3.zip › raw data for Figure 6/Figure 6 A/LPS+Fer-1.jpg]

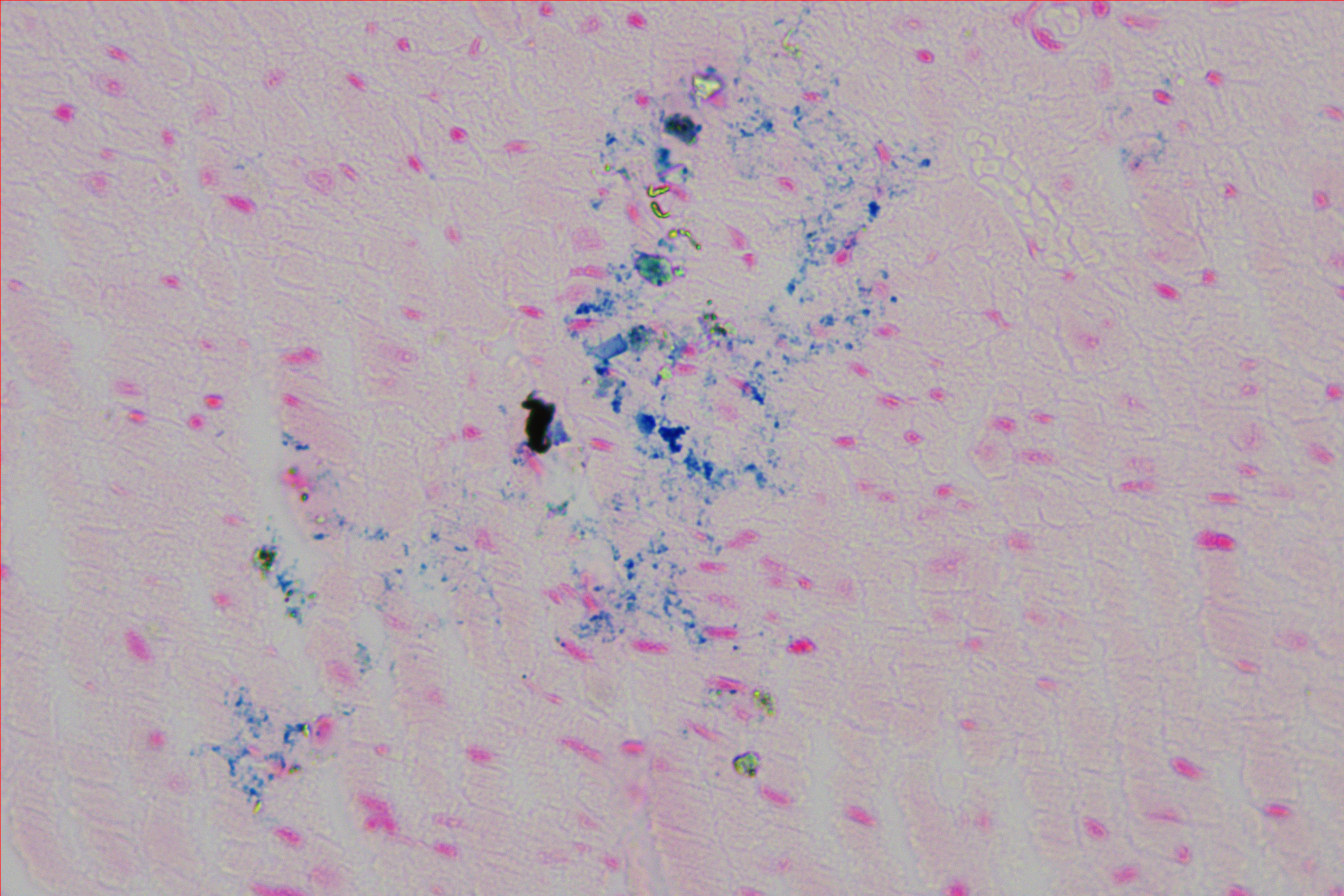

Supplement: Supplementary file 3 [file Datasheet3.zip › raw data for Figure 6/Figure 6 A/LPS.jpg]

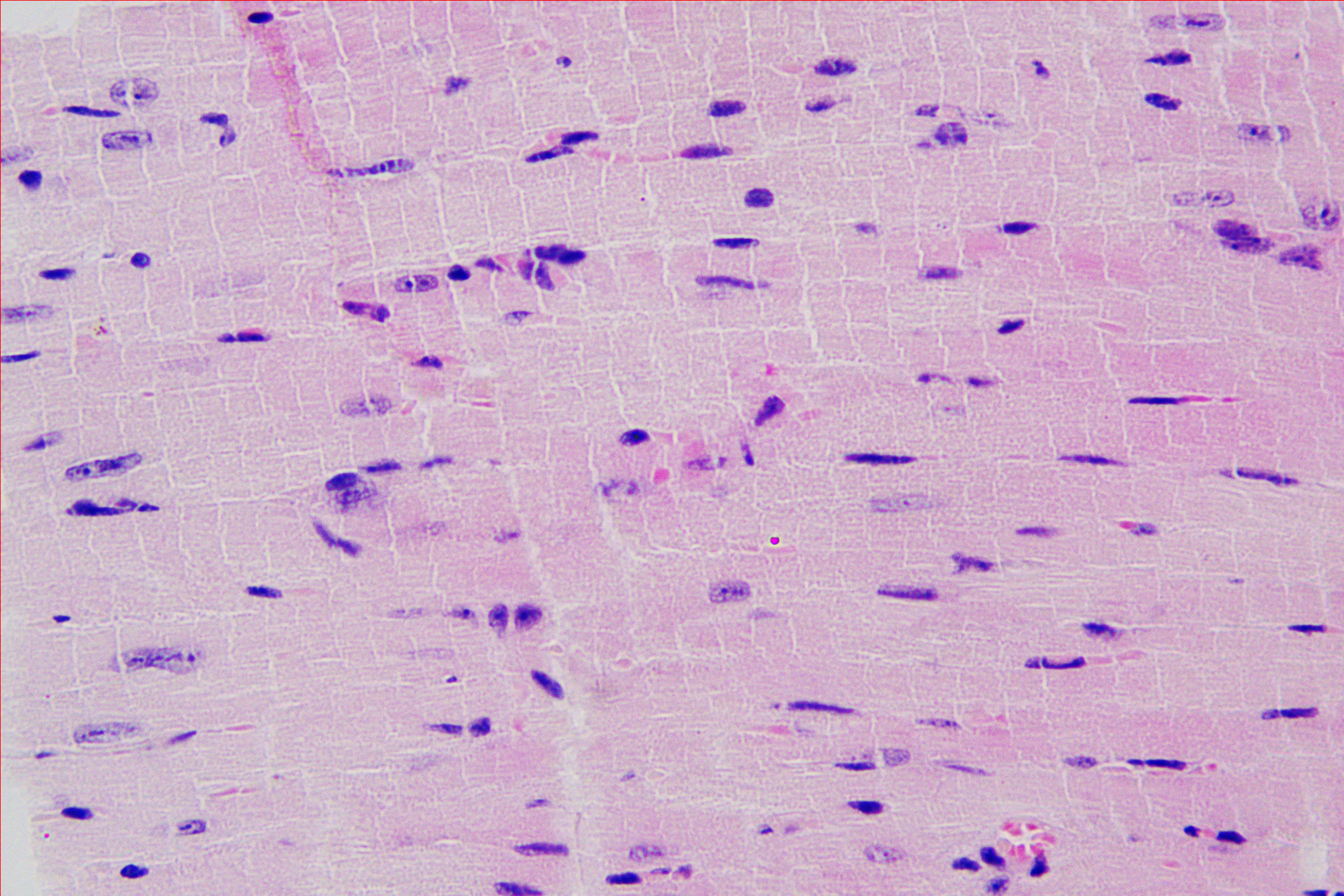

Supplement: Supplementary file 3 [file Datasheet3.zip › raw data for Figure 6/Figure 6 B/Control-1.jpg]

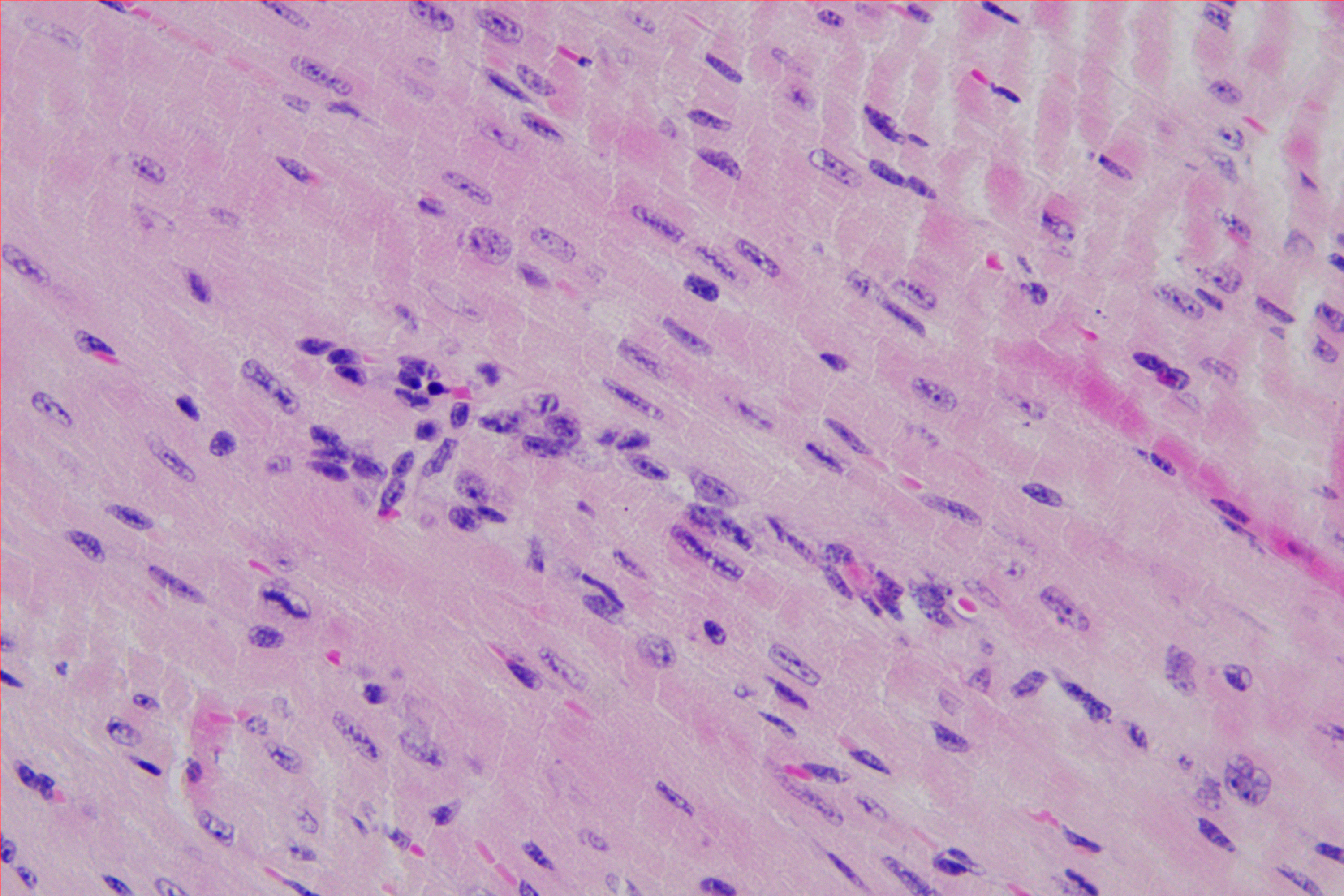

Supplement: Supplementary file 3 [file Datasheet3.zip › raw data for Figure 6/Figure 6 B/LPS+Fer-1-1.jpg]

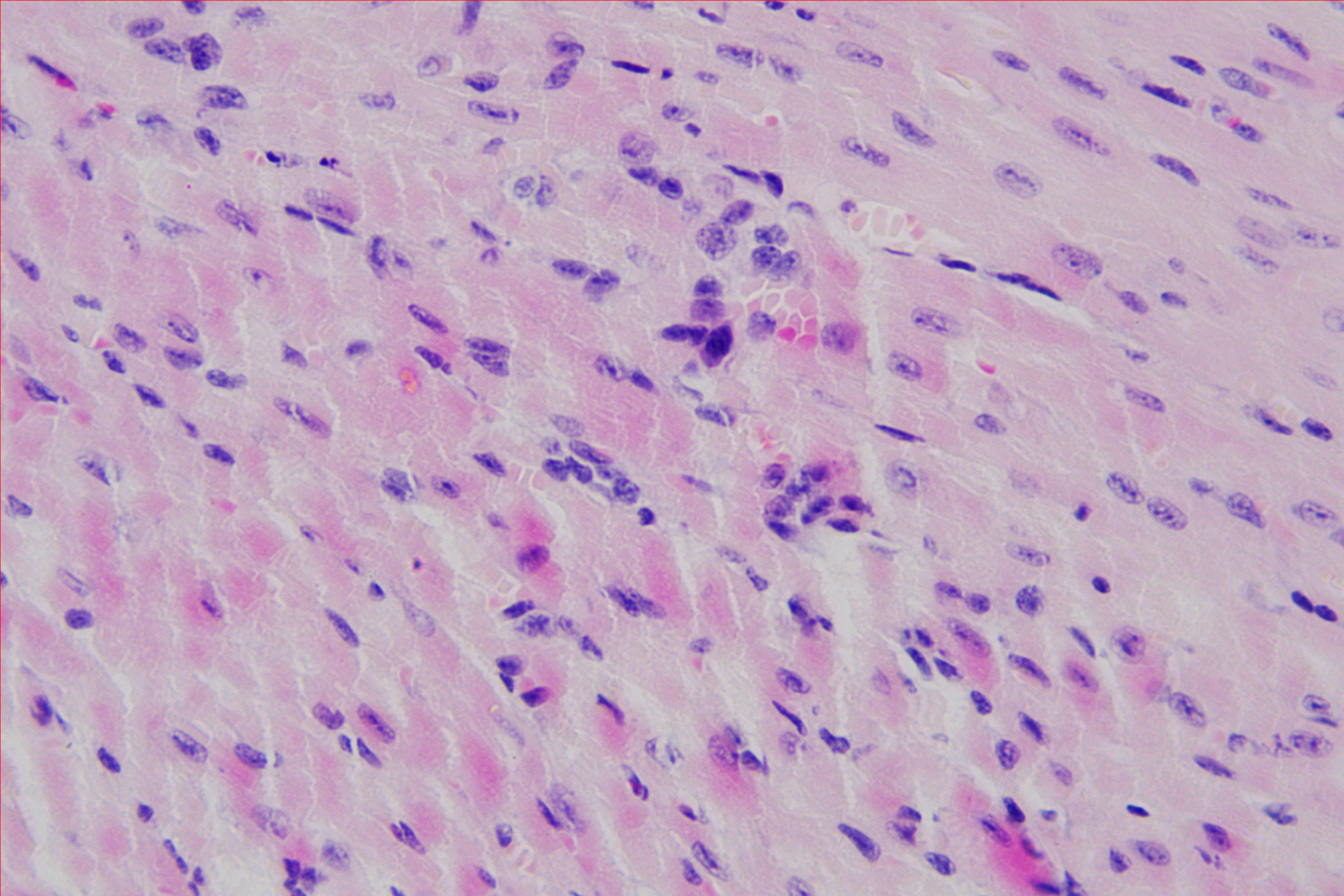

Supplement: Supplementary file 3 [file Datasheet3.zip › raw data for Figure 6/Figure 6 B/LPS-1.jpg]

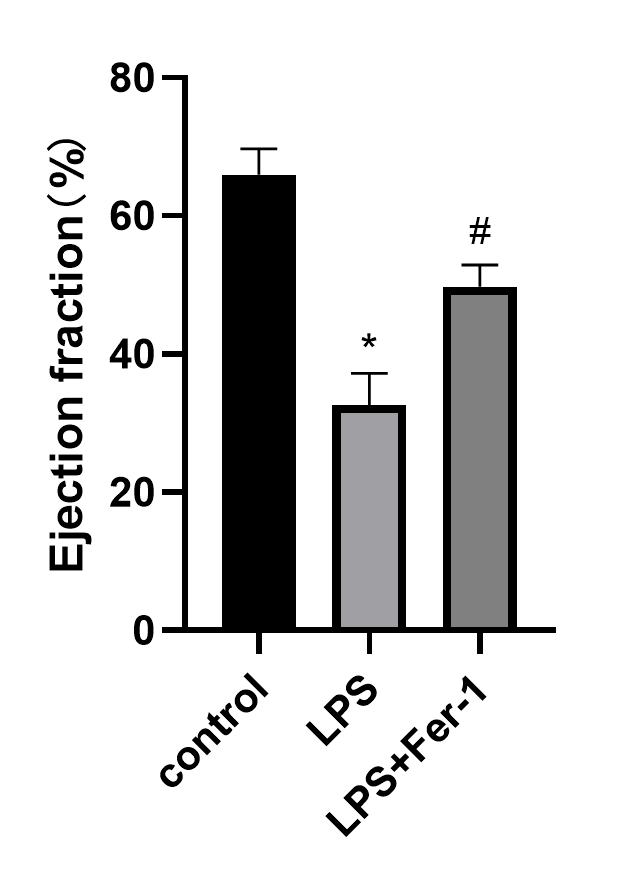

Supplement: Supplementary file 3 [file Datasheet3.zip › raw data for Figure 6/Figure 6 C&D&E(echocardiogram)/EF%.png]

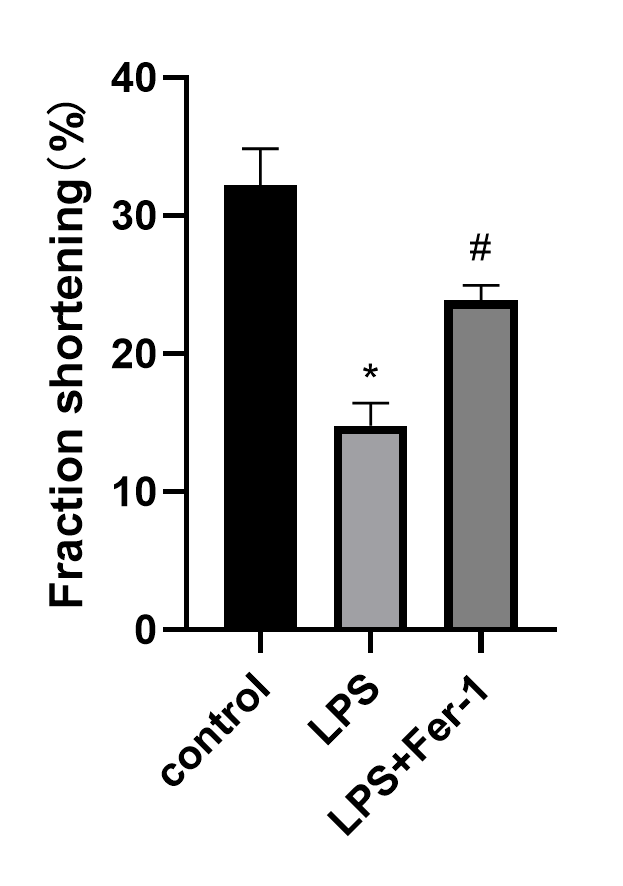

Supplement: Supplementary file 3 [file Datasheet3.zip › raw data for Figure 6/Figure 6 C&D&E(echocardiogram)/FS%.png]

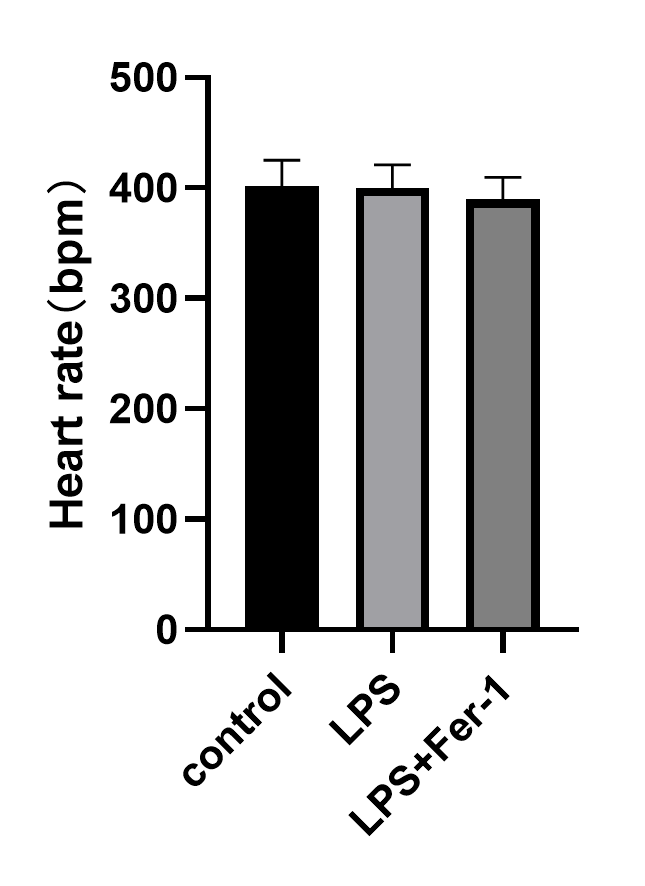

Supplement: Supplementary file 3 [file Datasheet3.zip › raw data for Figure 6/Figure 6 C&D&E(echocardiogram)/HR(bpm).png]

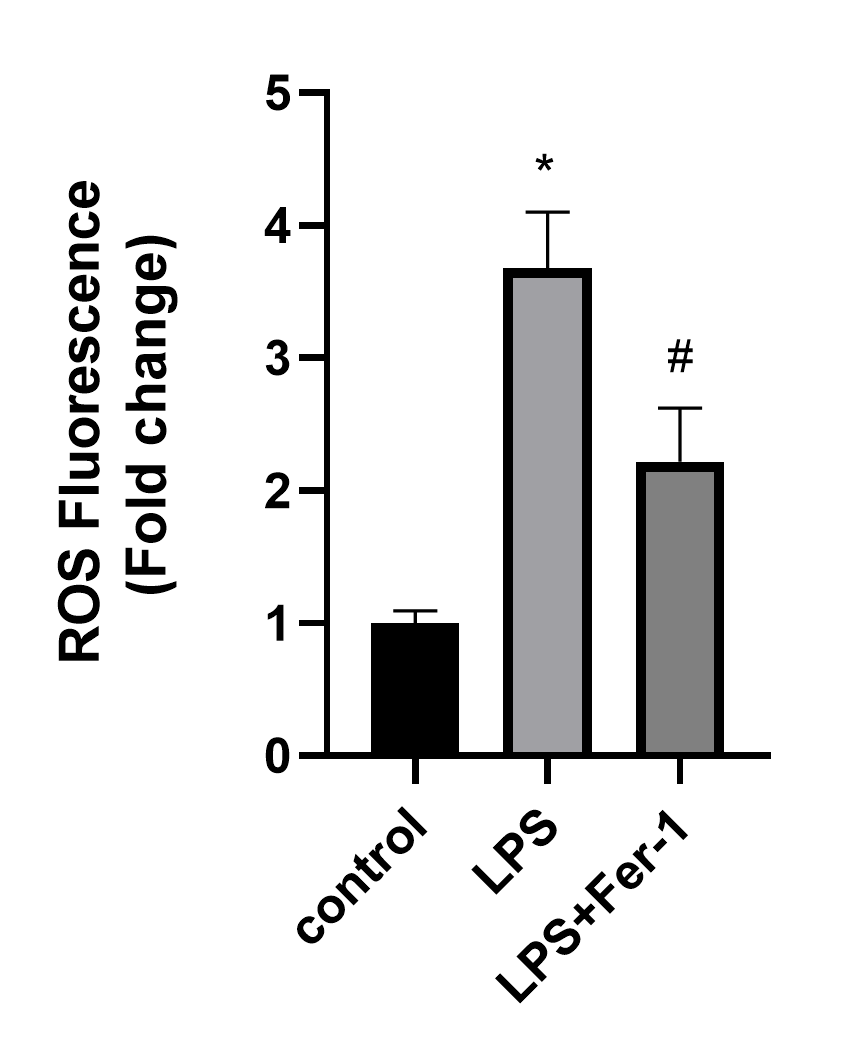

Supplement: Supplementary file 3 [file Datasheet3.zip › raw data for Figure 6/Figure 6 F/ROS Fluorescence.png]

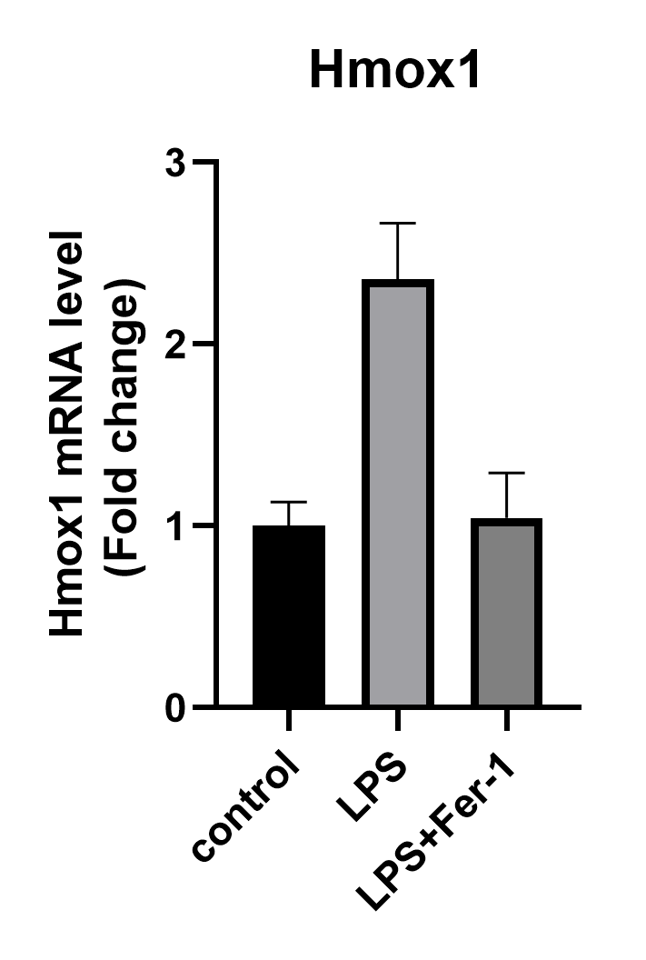

Supplement: Supplementary file 3 [file Datasheet3.zip › raw data for Figure 6/Figure 6 G&H&I (RT-qPCR)/Hmox1.png]

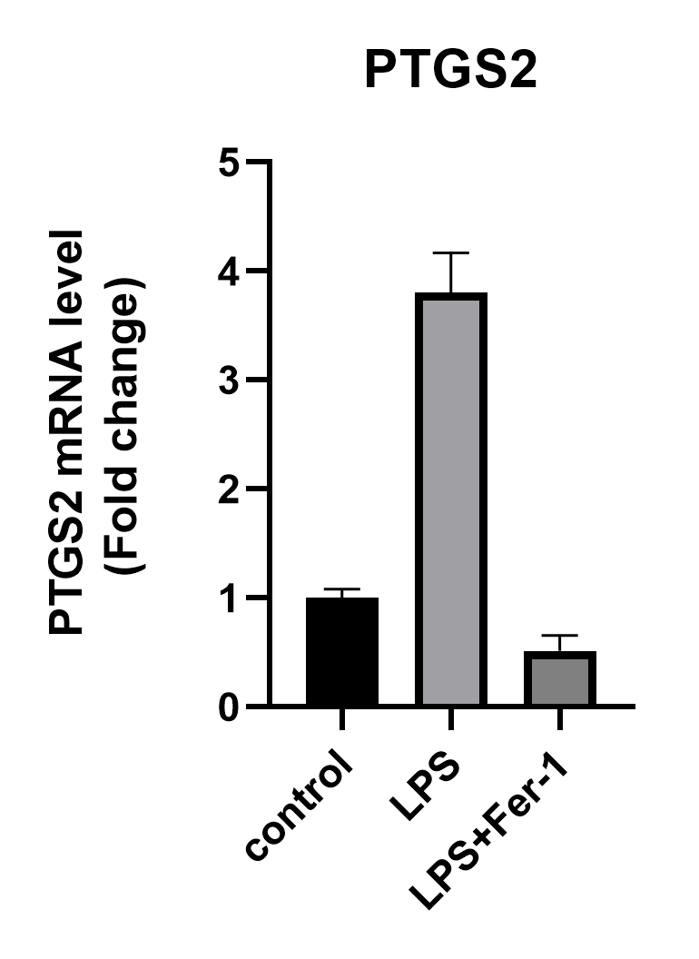

Supplement: Supplementary file 3 [file Datasheet3.zip › raw data for Figure 6/Figure 6 G&H&I (RT-qPCR)/PTGS2.png]

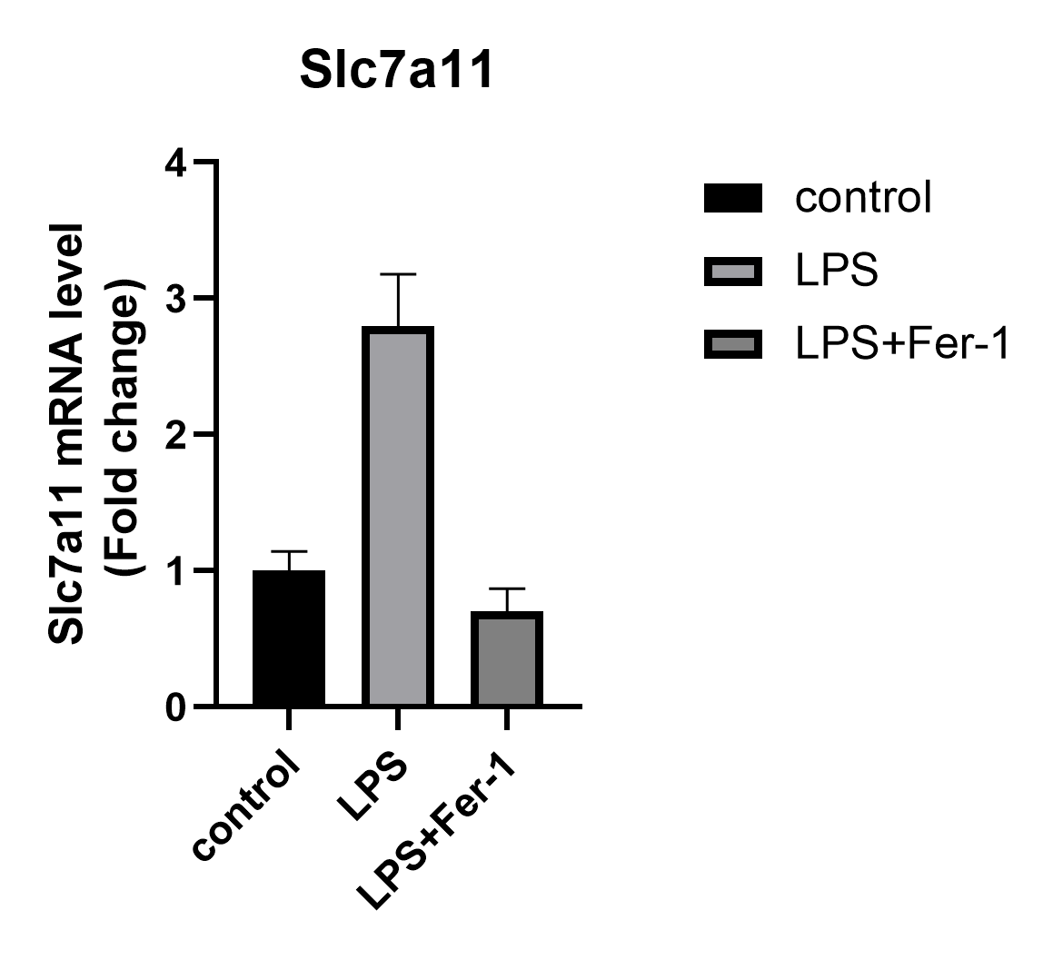

Supplement: Supplementary file 3 [file Datasheet3.zip › raw data for Figure 6/Figure 6 G&H&I (RT-qPCR)/Slc7a11.png]

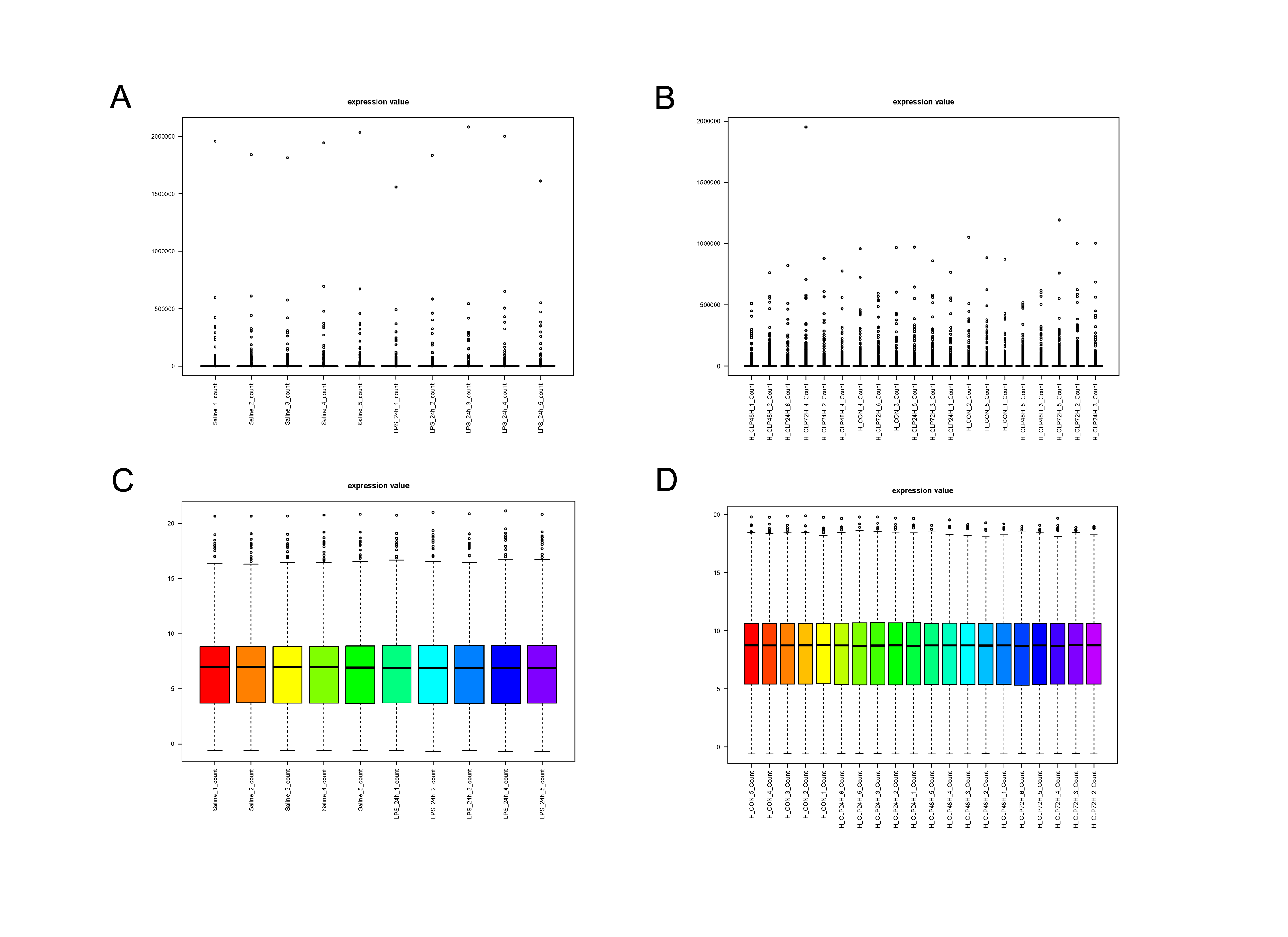

Supplement: Supplementary file 4 [file Image1.tif]

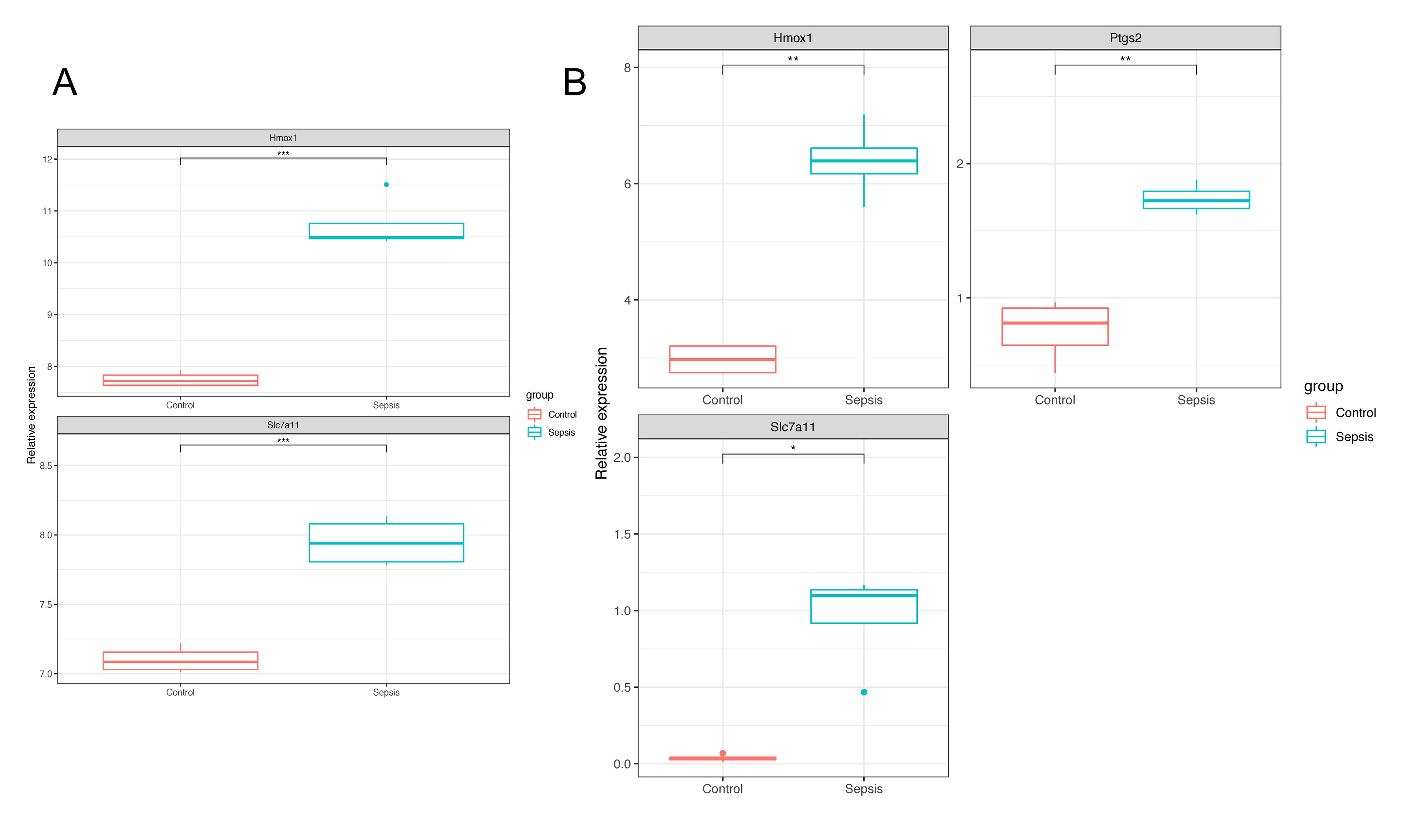

Supplement: Supplementary file 5 [file Image2.tif]

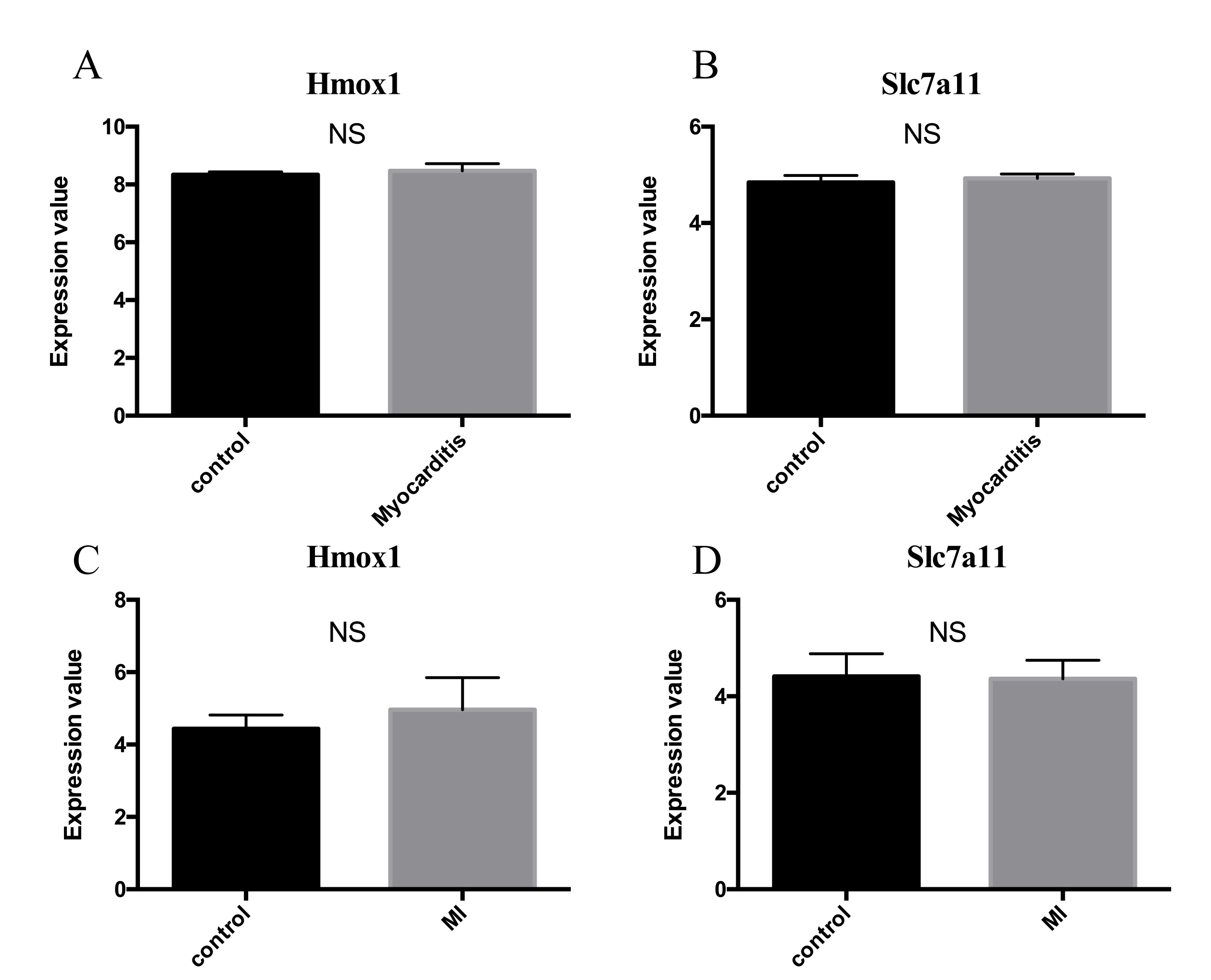

Supplement: Supplementary file 6 [file Image3.tif]
